# A nine–consensus–prognostic –gene–based prognostic signature, recognizing the dichotomized subgroups of gastric cancer patients with different clinical outcomes and therapeutic strategies

**DOI:** 10.3389/fgene.2022.909175

**Published:** 2022-09-26

**Authors:** Dan Ji, Yang Yang, Fei Zhou, Chao Li

**Affiliations:** ^1^ Department of Basic Medicine, Anhui Medical College, Hefei, Anhui, China; ^2^ Huangshan Health Vocational College, Huangshan, Anhui, China; ^3^ Department of General Surgery, Hefei First People’s Hospital, Hefei, China

**Keywords:** immunotherapy, prognostic signature, gastric cancer, chemothearpy, validation

## Abstract

**Background:** The increasing prevalence and mortality of gastric cancer (GC) has promoted the urgent need for prognostic signatures to predict the long-term risk and search for therapeutic biomarkers.

**Methods and materials:** A total of 921 GC patients from three GEO cohorts were enrolled in the current study. The GSE15459 and GSE62254 cohorts were used to select the top prognostic gene *via* the evaluation of the area under the receiver operating characteristic (ROC) curve (AUC) values. The GSE84437 cohort was used as the external validation cohort. Least absolute shrinkage and selector operation (LASSO) regression analysis was applied to reduce the feature dimension and construct the prognostic signature. Furthermore, a nomogram was constructed by integrating the independent prognostic analysis and validated by calibration plot, decision curve analysis and clinical impact curve. The molecular features and response to chemo-/immunotherapy among risk subgroups were evaluated by the “MOVICS” and “ESTAMATE” R packages and the SubMap algorithm. Lauren classification and ACRG molecular subtype were obtained to compare with the risk model.

**Results:** Forty-four prognosis-associated genes were identified with a preset cutoff AUC value of 0.65 in both the GSE62254 and GSE15459 cohorts. With the 10-fold cross validation analysis of LASSO, nine genes were selected to construct the nine-consensus-prognostic-gene signature. The signature showed good prognostic value in the GSE62254 (*p* < 0.001, HR: 3.81, 95% CI: 2.44–5.956) and GSE15459 (*p* < 0.001, HR: 2.65, 95% CI: 1.892–3.709) cohorts and the external validation GSE84437 cohort (*p* < 0.001, HR: 2.06, 95% CI: 1.554–2.735). The nomogram constructed based on two independent predictive factors, tumor stage and the signature, predicted events tightly consistent with the actual (Hosmer–Lemeshow *p* value: 1-year, 0.624; 3-years, 0.795; 5-years, 0.824). For the molecular features, we observed the activation of apical junction, epithelial mesenchymal transition, and immune pathways in the high-risk group, while in the low-risk group, cell cycle associated G2M, E2F and MYC target pathways were activated. Based on the results we obtained, we indicated that gastric patients in the low-risk group are more suitable for 5-fluorouracil therapy, while high-risk group patients are more suitable for anti-CTLA4 immunotherapy, these results need more support in the further studies. After compare with proposed molecular subtypes, we realized that the nine-consensus prognostic gene signature is a powerful addition to identify the gastric patients with poor prognosis.

**Conclusion:** In summary, we constructed a robust nine-consensus-prognostic-gene signature for the prediction of GC prognosis, which can also predict the personalized treatment of GC patients.

## Introduction

Gastric cancer (GC) is among the fifth most common malignancies worldwide, with almost 1000000 new cases recorded annually (Bray et al., 2018). The incidence rates of GC vary by country and region, and two-thirds are reported in developing countries (Thomassen et al., 2014). It was proposed that 80% of patients were diagnosed with GC at an advanced stage due to a lack of early characteristic complaints and signs, resulting in 800000 deaths each year, and is the third reason for cancer-specific death among all types of malignancies (Lou et al., 2021). Peritoneal dissemination is the widely accepted cause responsible for the high recurrence and dismal metastasis rates of GC (Thomassen et al., 2014). Once peritoneal dissemination onset occurs, most patients might suffer from bowel obstruction and the formation of massive malignant ascites and thus succumb within 4 months (Lee et al., 2021). Currently, multidisciplinary management strategies centered around surgical resection are the main treatments to decrease recurrence and delay metastasis for GC, including adjuvant therapy, radiotherapy, and targeted therapy (Lin Y. et al., 2021). However, numerous patients with GC relapse or develop metastatic disease even after radical resection. To optimize the therapeutic schemas for different patients, the tumor-node-metastasis (TNM) staging system is generally employed for risk stratification and prognostic prediction (Sasako et al., 2010). However, it was observed that patients with the same TNM stages presented different clinical outcomes (Tang et al., 2020). Fluoropyrimidine-platinum doubletHerein is the first-line agent in adjuvant therapy, while just 9% of patients estimated benefit from it (Bang et al., 2012). The pivotal reason responsible for the heterogeneity of prognosis and therapy is the different molecular features of each patient. Less attention has been given to the genetic alteration of GC in the past (Cristescu et al., 2015). Herein, further investigation of the underlying molecular mechanisms of GC and prognostic and therapeutic biomarkers are imperative.

The genomic landscape of GS has been revealed by large-scale next-generation sequencing analysis in decades, and comprehensive molecular alterations in GS have been reported, such as PIK3CA mutation, DNA hypermethylation, and amplification of JAK2, CD274 and PDCD1G2. Different genetic alterations were validated to correlate with significantly different clinicopathological features. Limitations exist in the small cohorts and incomplete analysis, which hinders its use in clinical settings (Tay et al., 2003; Cho et al., 2011; The Cancer Genome Atlas Research Network, 2014; Cristescu et al., 2015). GC with HER2 amplification was proven to be sensitive to trastuzumab, while a small proportion of patients would benefit from it (Bang et al., 2010). Microsatellite instability (MSI) is one subtype defined by TCGA. A recently published study proved that extra chemotherapy has little benefit compared to surgery alone. Regrettably, it can help few patients because 5%–10% of GC cases were divided into the MSI subgroup among the four molecular subtypes defined by TCGA (The Cancer Genome Atlas Research Network, 2014; Sohn et al., 2017). For immunology in GC, MSI, and PD-L1 are generally employed to guide clinical decision-making; the problem is that their modest predictive capability only takes limited help for clinicians (Keenan et al., 2019). Therefore, further research in this field is urgently needed.

In the present study, we identified nine prognosis-related genes of GC through ROC and LAASO algorithms and established a prognostic signature for risk stratification. We tested the discriminative accuracy and reliability of this model *via* robust statistical methods and validated that this signature served as an independent risk factor in GC. A nomogram risk model was established to facilitate risk stratification in a quantitative way. The accuracy and clinical value of this nomogram were confirmed with calibration and clinical decision curve analysis. In addition, we comprehensively elucidated different signaling activation statuses and immunocyte infiltration landscapes among the high- and low-risk groups.

## Methods and materials

### Cohort summary

Four GC cohorts from GEO were enrolled for the current study, including 300 patients from the GSE62254 cohort, 190 patients from the GSE15459 cohort, 431 patients from the GSE84437 cohort and 294 patients from TCGA-STAD cohort. The clinical features of each cohort were also collected, including age, sex, and tumor stage. The overall survival (OS) status was regarded as the endpoint of the follow-up, and patients with a follow-up time less than one more were eliminated to ensure that the new findings were more stable and representative. Details of the enrolled cohorts are shown in Table 1.

**TABLE 1:** Clinical features for the enrolled three datasets.

	GSE15459 (N = 190)	GSE62254 (N = 300)	GSE84437 (N = 431)	TCGA-STAD (N = 294)	Overall (N = 1215)
OS					
Alive	95 (50.0%)	148 (49.3%)	224 (52.0%)	175 (59.5%)	642 (52.8%)
Dead	95 (50.0%)	152 (50.7%)	207 (48.0%)	119 (40.5%)	573 (47.2%)
OS time, months					
Mean (SD)	38.8 (43.3)	50.6 (31.4)	70.5 (47.2)	21.1 (18.4)	48.7 (42.1)
Median [Min, Max]	19.7 [0.200, 158]	57.9 [1.00, 106]	70.0 [1.00, 161]	16.8 [0.525, 122]	33.0 [0.200, 161]
Age, years					
Mean (SD)	64.3 (13.2)	61.9 (11.4)	60.0 (11.6)	65.1 (10.1)	62.4 (11.6)
Median [Min, Max]	66.5 [23.4, 92.4]	64.0 [24.0, 86.0]	62.0 [27.0, 86.0]	67.0 [35.0, 86.0]	64.0 [23.4, 92.4]
Gender					
Female	67 (35.3%)	101 (33.7%)	137 (31.8%)	101 (34.4%)	406 (33.4%)
Male	123 (64.7%)	199 (66.3%)	294 (68.2%)	193 (65.6%)	809 (66.6%)
Stage					
Stage I	31 (16.3%)	30 (10.0%)	21 (4.9%)	41 (13.9%)	123 (10.1%)
Stage II	29 (15.3%)	97 (32.3%)	138 (32.0%)	85 (28.9%)	349 (28.7%)
Stage III	71 (37.4%)	96 (32.0%)	272 (63.1%)	128 (43.5%)	567 (46.7%)
Stage IV	59 (31.1%)	77 (25.7%)	0 (0%)	31 (10.5%)	167 (13.7%)
Missing	—	—	—	9 (3.1%)	9 (0.7%)

OS, overall survival.

### Data preprocessing

For the gene expression profile, the gene symbols were annotated according to the corresponding platform. GSE62254 and GSE15459 were all based on the GPL570 platform, while GSE84437 was based on the GPL6947 platform. The gene expression file was first checked, and the potential batch effect was removed by the “sva” package (Supplementary Figure S1) and further scaled to ensure that all the data were on a similar order of magnitude to ensure that the prognostic formula could be used in a consistent situation. The scaled gene expression value of GSE15459 ranged from −1.046 to 17.393, GSE6224 from 2.008 to 23.465, and GSE84437 from 1.478 to 20.205. A total of 20750 genes presented in all three enrolled cohorts were filtered for further subsequent analysis.

### Identifying consensus prognostic genes and constructing a prognostic model

We carried out receiver operating characteristic (ROC) curve analysis to evaluate the prognostic value of each gene in the GSE62254 and GSE15459 cohorts, while the area under the ROC curve (AUC) values were used for comparison. The process was performed by the “pROC” package. The preset cutoff value used to select the most prognostic gene was 0.65 in each cohort. Least absolute shrinkage and selector operation (LASSO) regression analysis was applied in several studies to construct the prognostic model, as well as in the current study. The “glmnet” R package completed a 10-fold cross validation analysis and revealed the minimum lambda to minimize the mean cross-validation error. The corresponding index for each selected gene was multiplied and added together to calculate the risk score for each patient. The protein levels of the nine genes were also determined through the Human Protein Atlas (https://www.proteinatlas.org/). We compared the IHC staining picture under the same antibody of each protein between normal stomach tissue and GC tissue.

### Establishment of a predictive nomogram

We first performed multivariate Cox regression analysis to identify independent factors, age, sex, and pathologic stage, and the signature was enrolled. Then, the nomogram was established based on the independent prognostic factors to integrate the predicted value *via* the “rms” and “regplot” R packages. To assess the accuracy and reliability, we performed a calibration plot, decision curve analysis (DCA) and clinical impact curve analysis.

### Molecular pathway variation and immune infiltration analysis

The “MOVICS” R package (Lu et al., 2020) was developed to identify the molecular subtypes and characterize the molecular features of tumors. We used it to generate the enrichment scores of 50 HALLMARK gene sets (Subramanian et al., 2005) and 28 immunocyte gene sets (Yoshihara et al., 2013) and visualized them with a heatmap. Gene set enrichment analysis (GSEA) also displayed different enrichment scores among risk subgroups, and the gene sets were derived from GO biological processes obtained from the Molecular Signatures Database (MSigDB, https://www.gsea-msigdb.org/gsea/msigdb/index.jsp). The overall immune infiltration status was evaluated through the ESTIMATE score, which was calculated by the “ESTIMATE” R package. Correlations between the risk score and infiltration ESTIMATE scores were calculated using the Spearman method.

### Target therapy prediction

The Genomics of Drug Sensitivity in Cancer (GDSC) database was used to predict the IC50 of cisplatin and 5-fluorouracil, which was calculated based on 10-fold cross-validation ridge regression (Yang et al., 2013). Anti-immune checkpoint therapy has shown promise for malignancy treatment, especially anti-CTLA-4 and anti-PD-1/PD-L1 treatment. We downloaded the gene expression profile from a melanoma cohort that contained 47 cases who received immunotherapy and corresponding response information (McGranahan et al., 2016). The SubMap algorithm was used to predict the response to immunotherapy in the current study based on the similarity of expression profiles to reflect the treatment response (Hoshida et al., 2007; Lu et al., 2019; Meng et al., 2021).

### Statistical analysis

The T test or the Mann–Whitney U test was applied to assess differences between subgroups with continuous variables. Survival curves were drawn by the K-M method and log-rank test, and hazard ratio (HR) and 95% confidence interval (CI) values of candidate genes were calculated by a univariate Cox regression model. Multivariate Cox analysis was used to explore the independent prognostic effect of the risk score after adjustment for several clinical characteristics. With *p* < 0.05 as the standard of significance, a two-sided statistical test was used. R software (4.1.2) was used for all analyses.

## Results

### Identifying 44 prognostic genes and constructing the nine-consensus-prognostic-gene signature

**FIGURE 1:**
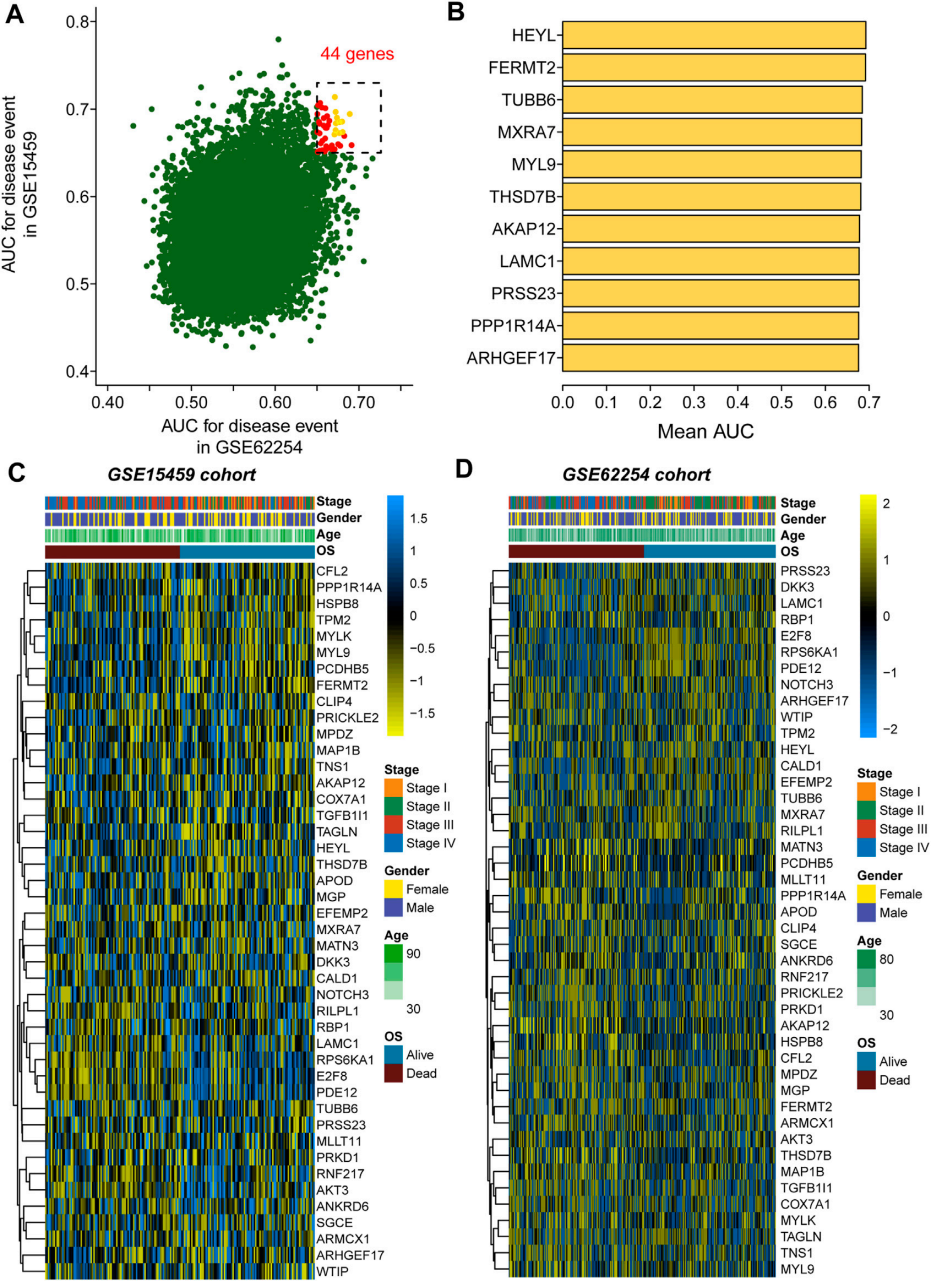
Selection of the 44 prognostic genes in GC patients. **(A)**, Scatter plot showing the prognostic value of genes in both the GSE62254 and GSE15459 cohorts; **(B)**, Eleven genes with the top average AUC values; **(C)**, Heatmap showing the expression of 44 selected genes in the GSE15459 cohort; **(D)**, Heatmap showing the expression of 44 selected genes in the GSE62254 cohort.

As mentioned in the methods section, we first evaluated the prognostic value of 20750 genes in both GSE62254 and GSE15459 (Figure 1A), and 44 genes were selected for subsequent analysis with a preset cutoff AUC value of 0.65. The top 10 prognostic genes included HEYL, FERMT2, TUBB6, MXRA7, MYL9, THSD7B, AKAP12, LAMC1, PRSS23, PPP1R14A and ARHGEF17 (Figure 1B). The heatmaps illustrate the expression value of these 44 genes among each patient in the GSE15459 cohort (Figure 1C) and GSE62254 cohort (Figure 1D).

**FIGURE 2:**
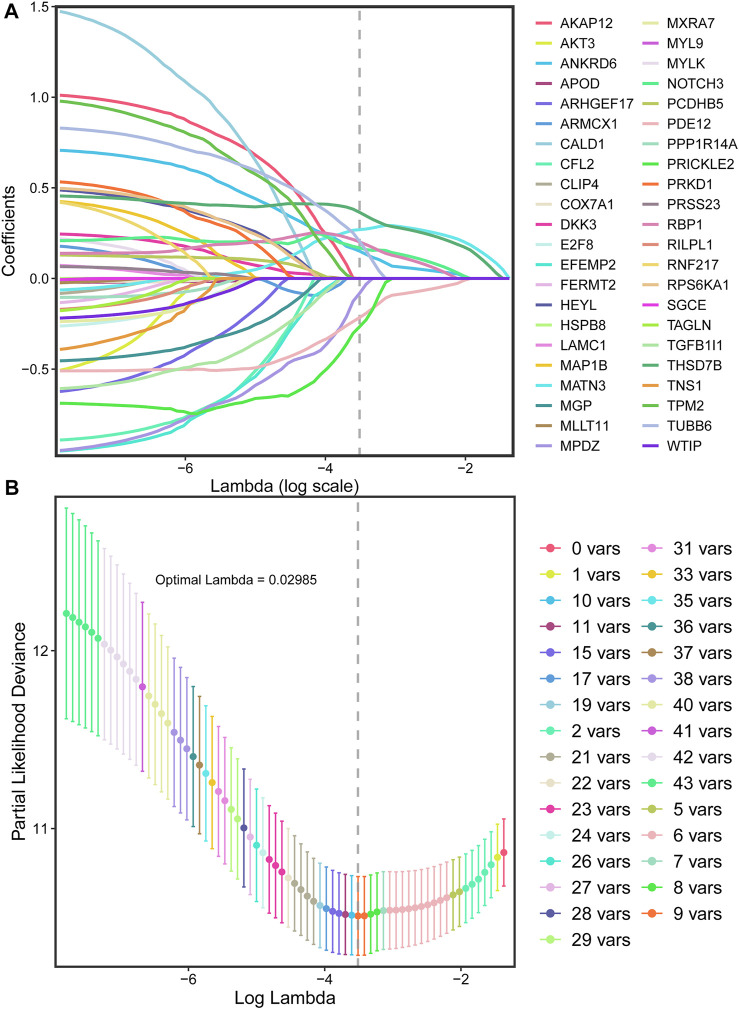
LASSO regression for the selection of prognostic genes. **(A)**, Optimal (minimum) lambda selection for overall survival in the LASSO regression model; **(B)**, LASSO coefficient profiles of variables selected for GC patients’ overall survival.

**FIGURE 3:**
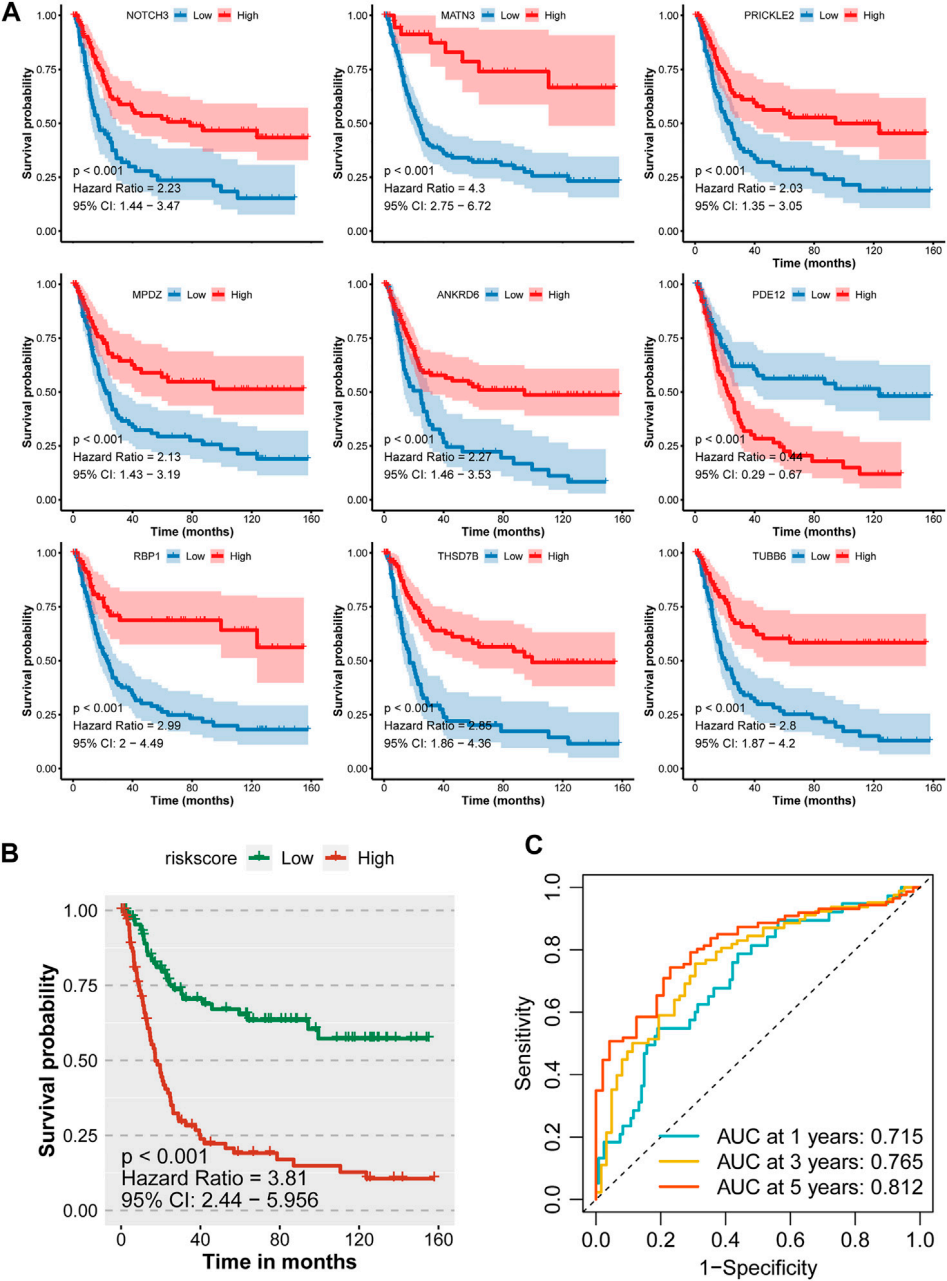
Prognostic value of the nine-consensus prognostic gene signature in the GSE15459 cohort. **(A)**, K-M plot showing the prognostic value of the nine selected genes; **(B)**, Kaplan–Meier curves for overall survival time of patients in the GSE15459 cohort. High-risk and low-risk groups were separated by the median value of risk score; **(C)**, Time-dependent ROC curves showing the predictive efficiency of the risk signature for GC patients in the GSE15459 cohort.

With the 10-fold cross validation analysis of LASSO, we revealed a minimal lambda value of 0.02985 (Figures 2A,B) and identified a nine-consensus-prognostic-gene signature, including NOTCH3 (*p* = 0.001, HR: 1.99, 95% CI: 1.33–2.98), MATN3 (*p* < 0.001, HR: 2.40, 95% CI: 1.60–3.61), PRICKLE2 (*p* = 0.004, HR: 1.82, 95% CI: 1.22–2.73), MPDZ (*p* < 0.001, HR: 2.08, 95% CI: 1.39–3.12), ANKRD6 (*p* = 0.016, HR: 1.65, 95% CI: 1.10–2.46), PDE12 (*p* = 0.004, HR: 0.55, 95% CI: 0.37–0.82), RBP1 (*p* < 0.001, HR: 2.20, 95% CI: 1.47–3.29), TBSD7B (*p* < 0.001, HR: The risk score of the nine-consensus-prognostic-gene signature was calculated by the formula: risk score = 0.169*NOTCH3–0.082*MPDZ + 0.202*RBP1 + 0.269*MATN3 + 0.151*ANKRD6 + 0.365*THSD7B–0.266*PRICKLE2 – 0.216*PDE12 + 0.205*TUBB6. After separating the patients into low-risk and high-risk subgroups by the median value of the risk score, we observed a dramatic OS difference, which also indicated the prognostic value of the signature (Figure 3B, *p* < 0.001, HR: 3.81, 95% CI: 2.44–5.956). In addition, the prognostic value also assessed by the ROC curve resulted in the preferable AUC values (Figure 3C, 1-year: 0.715,3-years: 0.765,5-years: 0.812).

**FIGURE 4:**
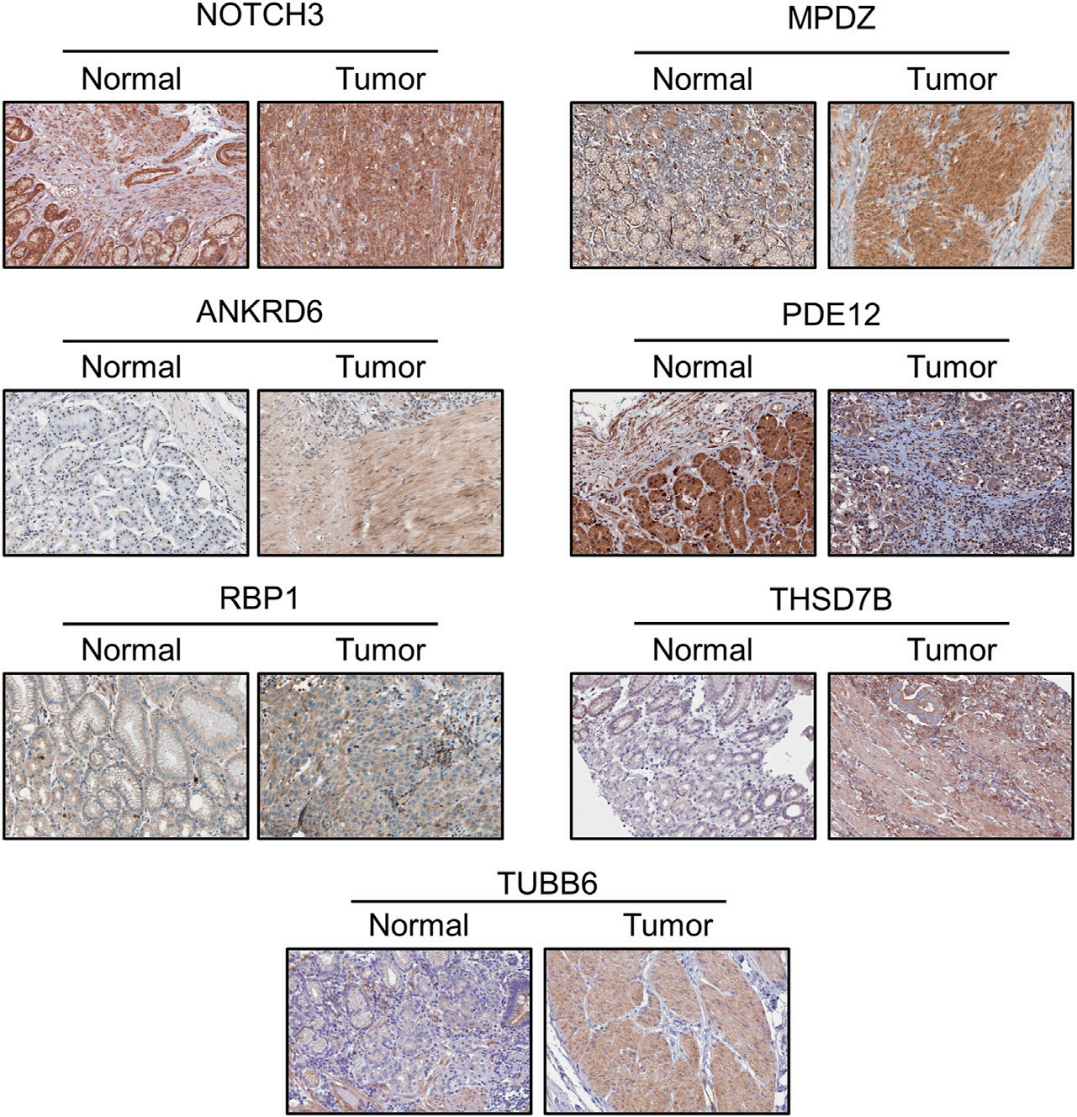
The protein level by IHC staining among normal stomach tissue and gastric cancer tissue.

The protein levels of the nine genes were also determined through the Human Protein Atlas (https://www.proteinatlas.org/). We compared the IHC staining picture under the same antibody of each protein between normal stomach tissue and GC tissue. The protein levels of MATN3 and PRIHKLE2 are lacking in the database. For the residual seven proteins, we observed strong positive staining of MPDZ, THSD7B and TUBB6 in the tumor tissue compared with the negative staining of these proteins in normal tissue, which is consistent with the abovementioned results that the decreased expression of these three genes is linked to poor prognosis. Weak positive staining of ANKRD6 was also present in tumor tissue compared with the negative staining in normal tissue, also consistent with the survival data. The protein levels of NOTCH3 and RBP1 showed similar results between normal and tumor tissues. The decreased level of PDE12 indicated the poor prognosis of GC patients, and we observed weaker PDE12 staining in tumor tissue (Figure 4).

### The nine-consensus-prognostic-gene signature is an independent predictive factor for GC patients

We observed that the distribution of tumor stage (*p* < 0.001) showed a difference between the high-risk and low-risk subgroups, but sex (*p* = 0.76) and age (*p* = 0.24) showed no difference (Supplementary Figure S2). To further evaluate the prognostic value of the nine-consensus-prognostic-gene signature, we performed multivariate Cox analysis, which also enrolled the clinical features of age, sex, and tumor stage.

**FIGURE 5:**
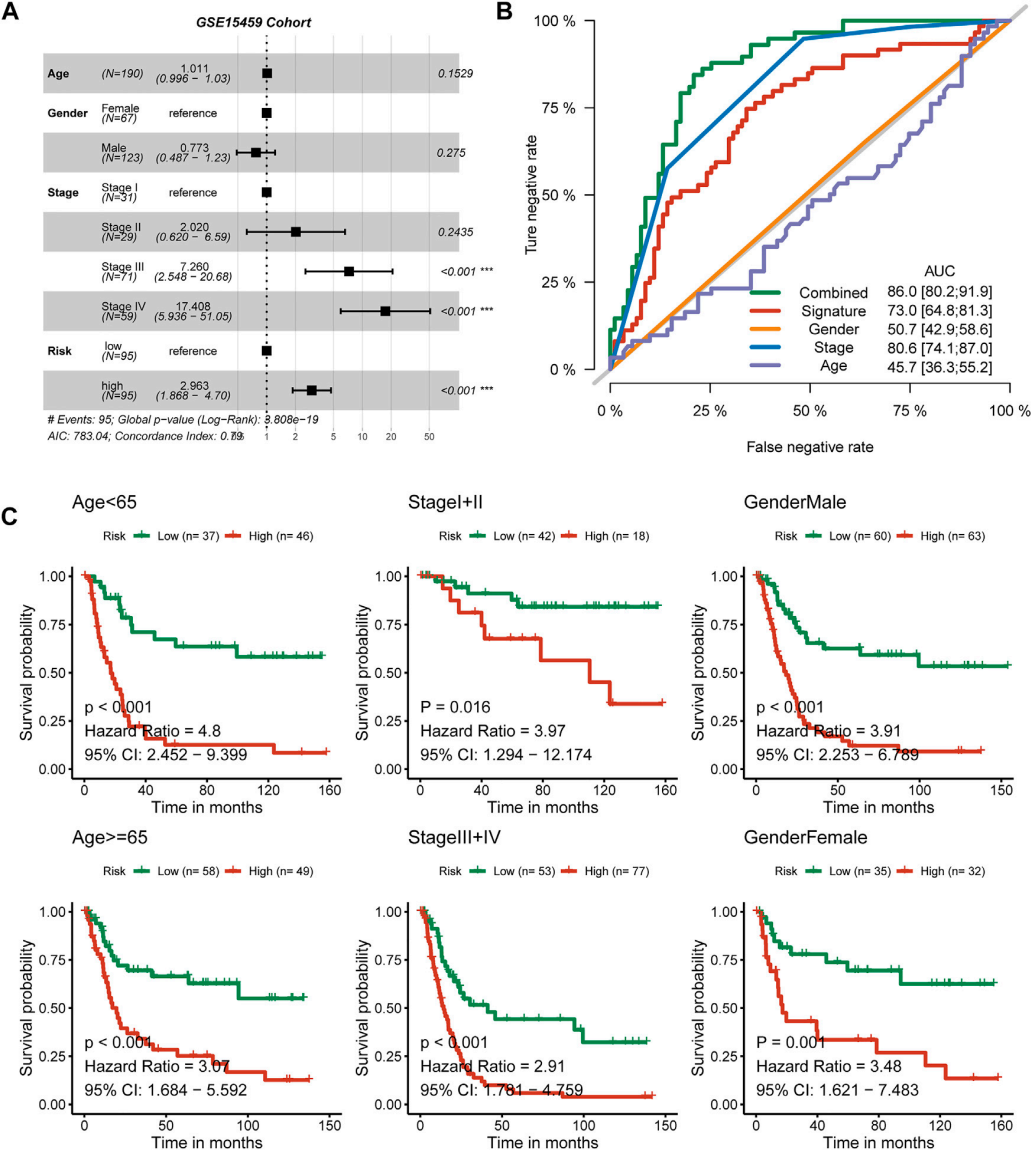
The nine-consensus-prognostic-gene signature is an independent prognostic factor for GC patients. **(A)**, Forest plot showing the hazard ratios from multivariate Cox regression analysis in the GSE15459 cohort; **(B)**, ROC curves showing the prognostic efficiency of the signature, clinical parameters and combined model; **(C)**, The prognostic value of the signature in clinical subgroups.

We observed that stage III was a risk factor for OS (*p* < 0.001, HR: 7.26, 95% CI: 2.548–20.68), as well as stage IV (*p* < 0.001, HR: 17.408, 95% CI: 5.936–51.05). The signature is also an independent predictive factor; patients in the high-risk group met a 2.963-fold death risk compared with low-risk group patients, and the 95% CI ranged from 1.868 to 4.70 (Figure 5A). We used the ROC curve to evaluate the separated prognostic value of each factor as well as the combined model. Tumor stage could predict the OS of GC patients with good values (AUC: 0.806, 95% CI: 0.741–0.870), and the nine-consensus-prognostic-gene signature showed a preferable result (AUC: 0.730 95% CI: 0.648–0.813). After adding the clinical features to the overall combined model, the AUC value increased to 0.860, with a 95% CI of 0.802–0.919 (Figure 5B). We also assessed the prognostic value of the nine-consensus-prognostic-gene signature in the clinical subgroup and presented excellent results, including age < 65 years (*p* < 0.001, HR: 4.80, 95% CI: 2.452–9.399), ≥ 65 years (*p* < 0.001, HR: 3.07, 95% CI: 1.684–5.592), stage I + II disease (*p* = 0.016, HR: 3.97, 95% CI: 1.294–12.174), stage III + IV disease (*p* < 0.001, HR: 2.91, 95% CI: 1.781–4.759), male sex (*p* < 0.001, HR: 3.91, 95% CI: 2.253–6.789), and female sex (*p* = 0.001, HR: 3.48, 95% CI: 1.621–7.483) (Figure 5C).

### Tumor stage and signature-based nomogram shows a good result

**FIGURE 6:**
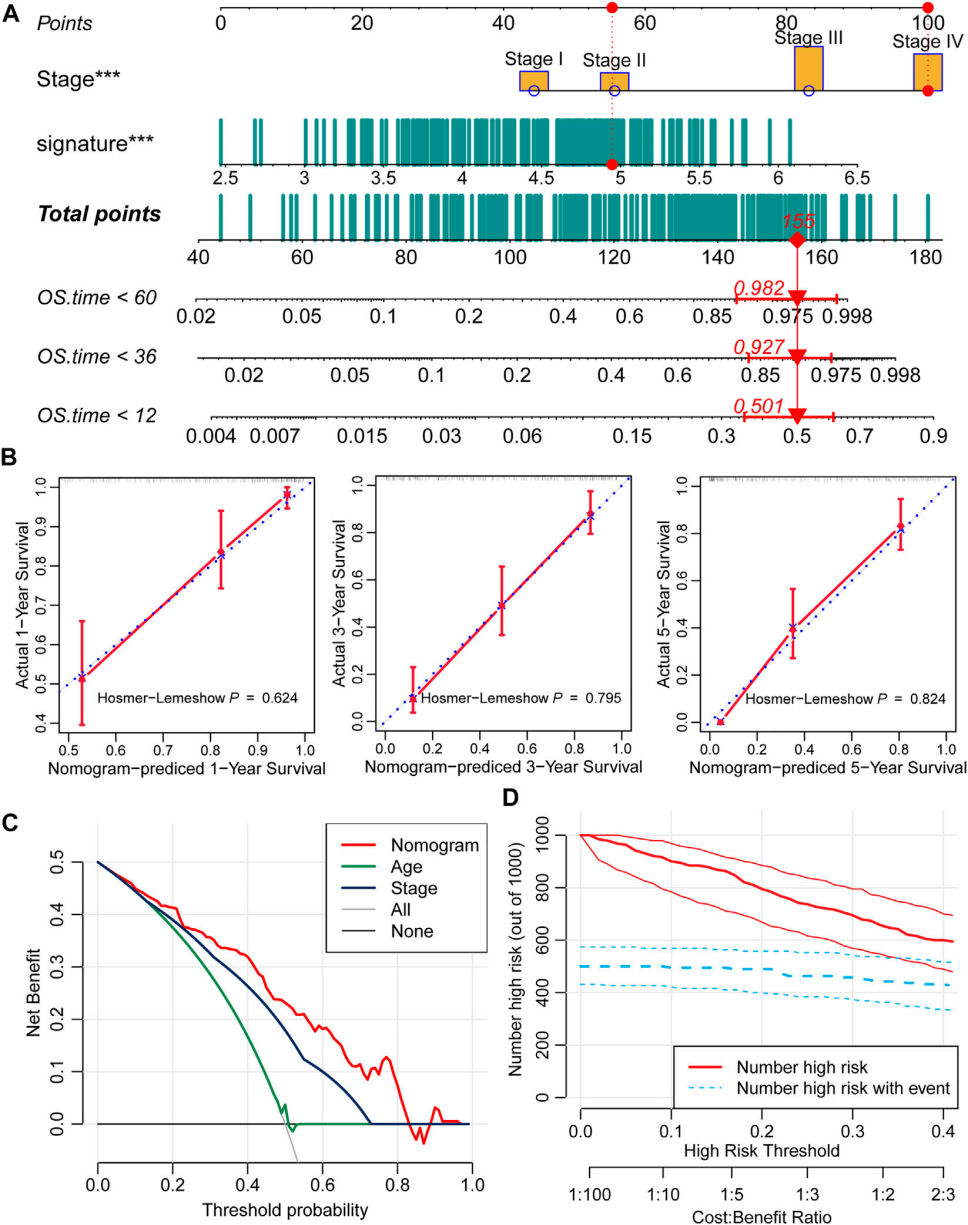
Prognostic nomogram based on the nine-consensus prognostic gene signature and clinical features. **(A)**, Nomogram for predicting the probability of 1-, 3-, and 5-years mortality; **(B)**, Calibration plots of the nomogram for predicting the probability of 1-, 3-, and 5-years overall survival; **(C)**, DCA showing the performance of the nomogram and other clinical features for predicting overall survival; **(D)**, Clinical impact curves of the nomogram for predicting overall survival.

Moreover, we constructed a nomogram based on the two independent predictive factors, tumor stage and the nine-consensus-prognostic gene signature (Figure 6A). The red point in the nomogram is an example of how to use the nomogram. The patient’s tumor is at stage IV, and the risk score is approximately five; therefore, the total point is 155, indicating that the risk of death is 0.501 at 1 year, 0.927 at 3 years, and 0.982 at 5 years. The nomogram-predicted results of the events were consistent with the actual results, as shown in the calibration curve of 1-year, 3-years and 5-years, and along with the *p* values all higher than 0.05 calculated by the Hosmer–Lemeshow analysis (Figure 6B, 1-year: *p* = 0.624,3-years: *p* = 0.795 and 5-years: *p* = 0.824).

DCA calculated the net benefit to evaluate the clinical utility of the nomogram, and the results showed that in the broad threshold of OS (10%–80%), the clinical net benefit of the nomogram was greater than that of age or stage (Figure 6C). Clinical impact curves of the nomogram to predict OS showed great prediction abilities when the risk threshold was less than 0.4 (Figure 6D).

### Recognizing the molecular pathways and precision therapeutic strategy

**FIGURE 7:**
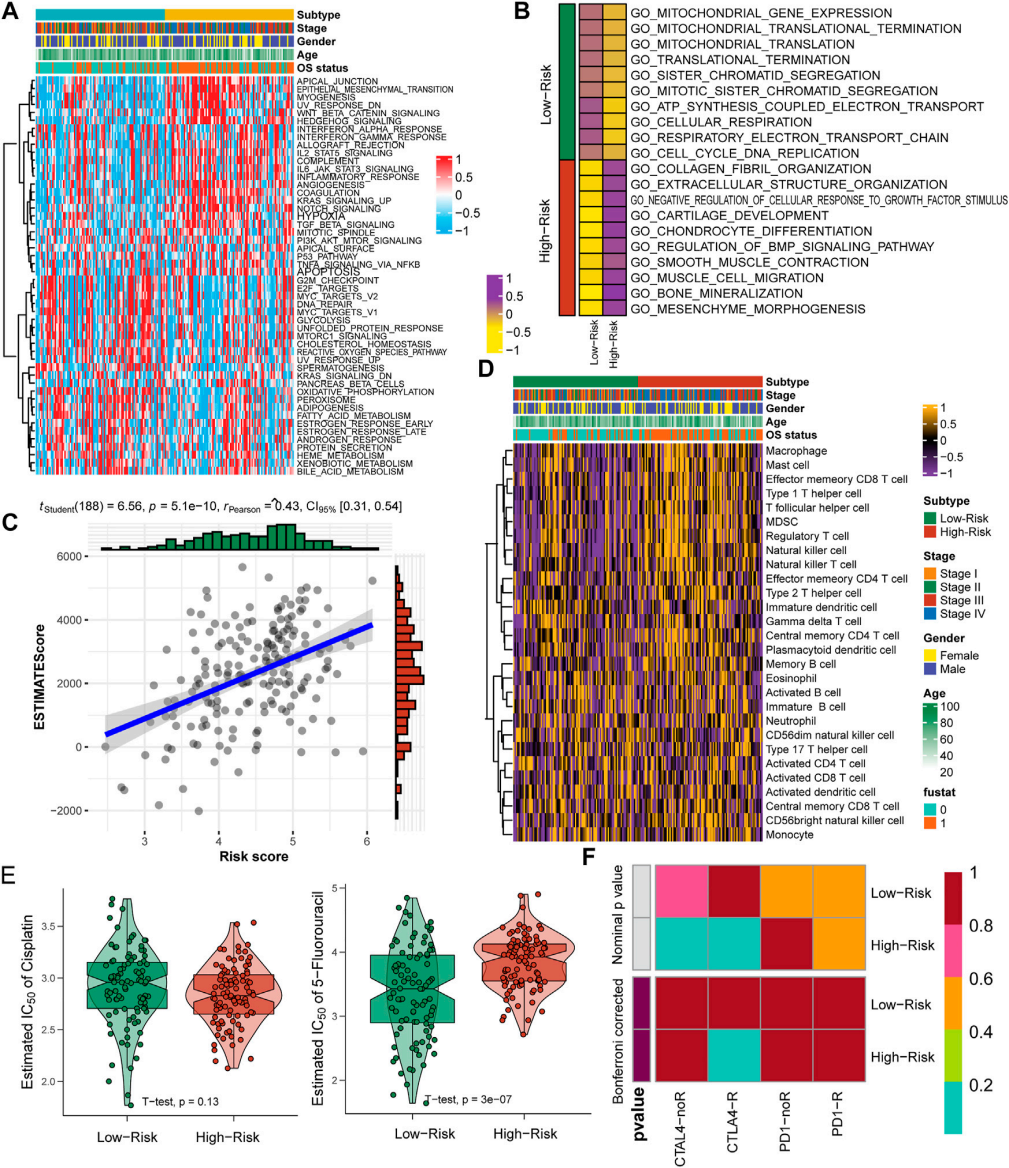
Specific molecular features and suitable treatment strategies for low-risk and high-risk GC patients. **(A)**, Heatmap showing the enrichment of 50 HALLMARK tumor pathways; **(B)**, Enrichment of GO terms in two risk groups; **(C)**, Correlation between nine-consensus-prognostic-gene signature generated risk score and the tumor infiltration ESTIMATE score; **(D)**, Heatmap showing the infiltration of 28 immunocyte gene sets; **(E)**, Estimated IC50 of cisplatin and 5-fluorouracil treatment in high-risk and low-risk subgroups; **(F)**, Submap analysis showing the potential response to anti-CTAL4 and anti-PD-1 therapy in high-risk and low-risk subgroups.

Tumor heterogeneity is impacted by diverse inner activated signaling pathways and always leads to different clinical outcomes and is important to guide the selection of appropriate therapeutic strategies. We assessed the signaling pathway activation of 50 HALLMARK typical tumor pathways and observed that patients in the high-risk group contained the activation pathways of apical junction, epithelial mesenchymal transition, WNT/beta-catenin signaling, as well as immune-associated pathways, including IL2/STAT5, IL6/JAK/STAT3, Notch signaling and the inflammatory response pathway (Figure 7A, Supplementary Table S1). For the low-risk group, cell cycle-associated G2 M, E2F and MYC target pathways were activated, as well as the oxidative phosphorylation and hormone response pathways (Figure 7A, Supplementary Table S1). The enrichment among GO terms also displayed similar results: the high-risk group enriched the pathways of collagen fibril and extracellular structure organization and mesenchyme morphogenesis, while the low-risk group enriched the pathways of mitochondrial gene expression and translation, ATP synthesis coupled electron transport and respiratory electron transport chain (Figure 7B, Supplementary Tables S2, S3). We further evaluated the association between the nine-consensus-prognostic-gene signature and immune infiltration by the “ESTIMATE” R package and revealed that the increased risk score was tightly linked with the ESTIMATE score, which indicated that the higher the risk score was, the higher the immune infiltration (R_Pearson_ = 0.43, *p* < 0.001, Figure 7C). The detailed infiltration score of 28 immunocytes estimated by ssGSEA also revealed that the high-risk group contained higher infiltration of immunocytes, especially macrophages, mast cells, type 1 T helper cells, regulatory T cells, natural killer cells and natural killer T cells (Figure 7D, Supplementary Table S4). To guide clinical treatment, we evaluated the response to cisplatin and 5-fluorouracil treatment and revealed that patients in the low-risk group were more suitable for 5-fluorouracil therapy than patients in the high-risk group (*p* < 0.001, Figure 7E), while the treatment efficiency of cisplatin in both groups was similar (*p* = 0.130, Figure 7E). The high-risk group contained activated immune pathways and higher infiltration of immunocytes. We also revealed that high-risk group patients were more suitable for anti-CTLA4 immunotherapy but not anti-PD-1 therapy (Figure 7F).

### Validation of the nine-consensus prognostic gene signature in the GSE62254 and GSE84437 cohorts

**FIGURE 8:**
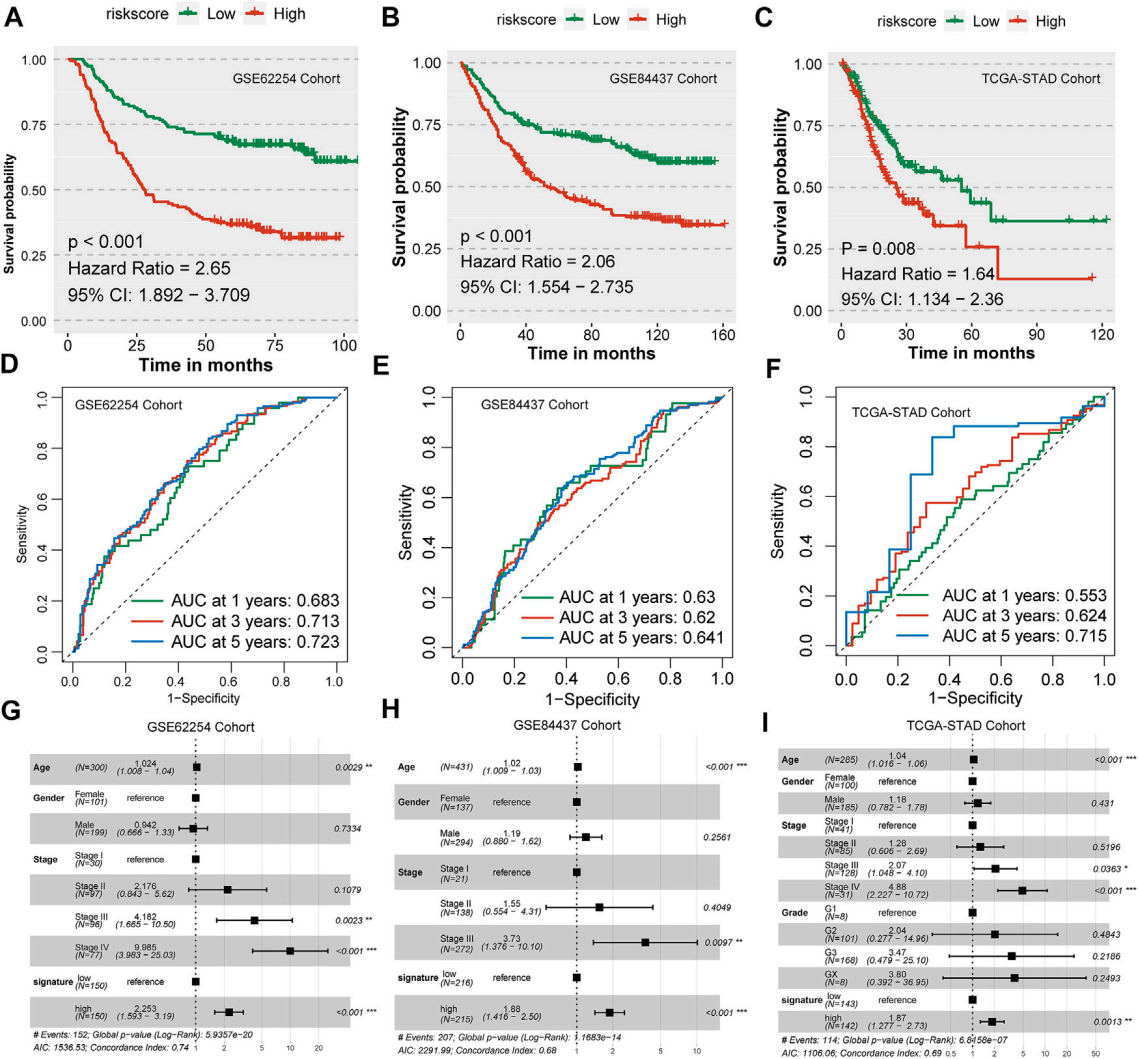
Validation of the nine-consensus-prognostic-gene signature in GSE62254, GSE84437 and TCGA-STAD cohorts. **(A)**, Kaplan–Meier curve for overall survival time of patients in the GSE62254 cohort; **(B)**, Kaplan–Meier curve for overall survival time of patients in the GSE84437 cohort; **(C)**, Kaplan–Meier curve for overall survival time of patients in the TCGA-STAD cohort; **(D)**, Time-dependent ROC curves showing the predictive efficiency of the risk signature in the GSE62254 cohort; **(E)**, Time-dependent ROC curves showing the predictive efficiency of the risk signature in the GSE84437 cohort; **(F)**, Time-dependent ROC curves showing the predictive efficiency of the risk signature in the TCGA-STAD cohort; **(G)**, Forest plot showing the hazard ratios from multivariate Cox regression analysis in the GSE62254 cohort; **(H)**, Forest plot showing the hazard ratios from multivariate Cox regression analysis in the GSE84437 cohort. **(I)**, Forest plot showing the hazard ratios from multivariate Cox regression analysis in the TCGA-STAD cohort.

First, we validated the prognostic efficiency of the nine-consensus-prognostic-gene signature in the GSE62254 cohort, which was used to select the 44 prognostic genes in the GSE15459 cohort. We first evaluated the prognostic value of the nine selected genes in the GSE62254 cohort, including NOTCH3 (*p* < 0.001, HR: 2.92, 95% CI: 2.10–4.07), MATN3 (*p* < 0.001, HR: 2.40, 95% CI: 1.63–3.53), PRICKLE2 (*p* < 0.001, HR: 2.39, 95% CI: 1.60–3.58), MPDZ (*p* < 0.001, HR: 2.35, 95% CI: 1.51–3.66), ANKRD6 (*p* < 0.001, HR: 2.40, 95% CI: 1.69–3.41), PDE12 (*p* < 0.001, HR: 0.44, 95% CI: 0.31–0.61), RBP1 (*p* < 0.001, HR: 2.66, 95% CI: 1.79–3.95), TBSD7B (*p* < 0.001, HR: 2.71, 95% CI: 1.78–4.12), TUBB6 (*p* < 0.001, HR: 2.39, 95% CI: 1.70–3.37) (Supplementary Figure S3). The risk score of each patient in the GSE62254 cohort was calculated along with the abovementioned formula, and we observed that the risk score could significantly separate GC patients into poor and favorable prognosis subgroups (*p* < 0.001, HR: 2.65, 95% CI: 1.892–3.709, Figure 8A). The prognostic AUC value was as high as 0.683 at 1 year, 0.713 at 3 years and 0.723 at 5 years (Figure 8D). The nine-consensus-prognostic-gene signature also acted as an independent prognostic factor in GSE62254 after adjusting for the clinical features of age, sex and stage (*p* < 0.001, HR: 2.253, 95% CI: 1.593–3.190, Figure 8G).

The validation of a prognostic signature in an external cohort that was not involved in the selection progress of the factors used to construct the model is essential; therefore, we further evaluated the prognostic value of the nine-consensus-prognostic-gene signature in the GSE84437 cohort. We also evaluated the prognostic value of the nine selected genes in the GSE84437 cohort, including NOTCH3 (*p* = 0.001, HR: 1.91, 95% CI: 1.38–2.64), MATN3 (*p* < 0.001, HR: 1.81, 95% CI: 1.32–2.47), PRICKLE2 (*p* < 0.001, HR: 1.88, 95% CI: 1.37–2.57), MPDZ (*p* < 0.001, HR: 1.82, 95% CI: 1.38–2.40), ANKRD6 (*p* = 0.002, HR: 1.67, 95% CI: 1.24–2.25), PDE12 (*p* < 0.001, HR: 0.60, 95% CI: 0.46–0.80), RBP1 (*p* = 0.003, HR: 1.80, 95% CI: 1.30–2.49), TBSD7B (*p* < 0.001, HR: 1.68, 95% CI: 1.27–2.22), and TUBB6 (*p* < 0.001, HR: 2.00, 95% CI: 1.44–2.77) (Supplementary Figure S4). Interestingly, we observed a separated prognosis in the risk score-separated high- and low-risk subgroups (*p* < 0.001, HR: 2.06, 95% CI: 1.554–2.735, Figure 8B). The prognostic AUC value was as high as 0.630 at 1 year, 0.620 at 3 years and 0.641 at 5 years (Figure 8E). The nine-consensus-prognostic-gene signature also acted as an independent prognostic factor in GSE84437 after adjusting for the clinical features of age, sex and stage (*p* < 0.001, HR: 1.880, 95% CI: 1.416–2.500, Figure 8H).

TCGA-STAD cohort was chosen as the second validation cohort. We calculated the risk score of each patient along with the formula and separated them as low-risk and high-risk subgroups with the median value. We observed that patients with high-risk in TCGA-STAD cohort showed a 1.64-fold of death than patients in low-risk group (*p* < 0.001, 95% CI: 1.134–2.360, Figure 8C), and the prognostic AUC value was as high as 0.715 at 5 years (Figure 8F). The nine-consensus-prognostic-gene signature also acted as an independent prognostic factor in GSE84437 after adjusting for the clinical features of age, sex and stage (*p* = 0.0013, HR: 1.870, 95% CI: 1.277–2.730, Figure 8I).

### Comparison between the nine-consensus prognostic gene signature and proposed molecular subtypes

**FIGURE 9:**
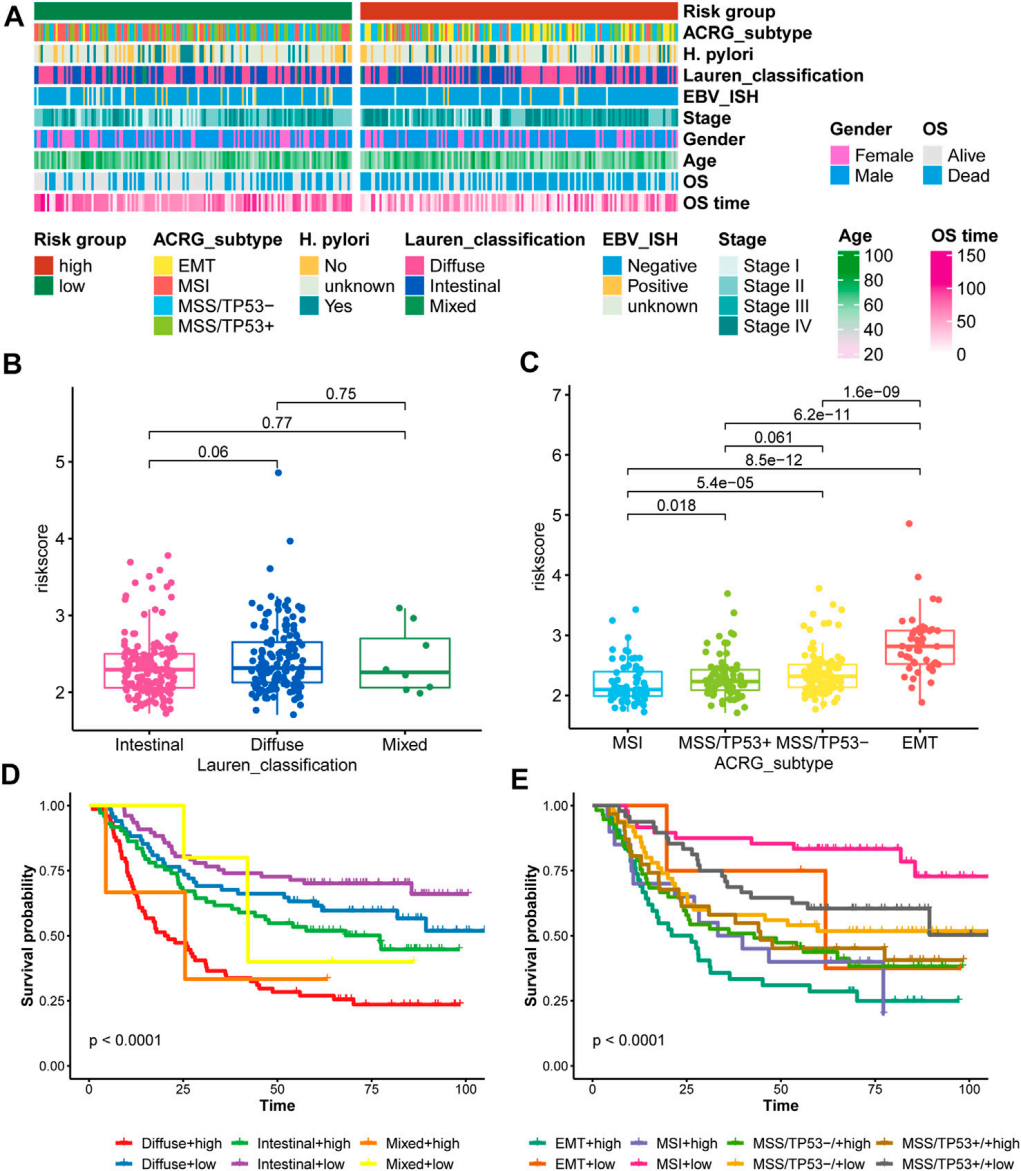
Comparison of the nine-consensus prognostic gene signature and proposed molecular subtypes. **(A)**, Heatmap showing the molecular features and clinical features in low- and high-risk subgroups; **(B)**, Box plot showing the comparison of risk score between Lauren classification subgroups; **(C)**, Box plot showing the comparison of risk score between ACRG subtypes; **(D)**, K-M plot showing the diverse overall survival between Lauren classification plus risk subgroups; **(E)**, K-M plot showing the diverse overall survival between ACRG subtypes plus risk subgroups.

For the ACRG/GSE62254 cohort, Crestescu et al. (Cristescu et al., 2015) reported the Lauren classification and pointed out the ACRG molecular subtype, we compared the contribution of the nine-consensus prognostic gene signature to the proposed molecular subtypes. The distribution of clinical features and molecular features showed in Figure 9A. We observed that more EMT subtypes in high-risk subgroup, as well as more Diffuse classification. Similarly, the average risk score in diffuse class is higher than that in intestinal class (Figure 9B, *p* = 0.06); EMT subtype contained the highest average risk score than the other three subtypes (Figure 9C, all *p* < 0.001). These results confirmed our risk subtyping to the prediction of prognosis, because that several studies already reported that the EMT activation or diffuse histology links with the poor prognosis of gastric cancer (Oh et al., 2018; Tanaka et al., 2021; Li Y. et al., 2022; Li M. et al., 2022). Moreover, we combined the risk subgroups with Lauren classification and ACRG molecular subtype. We found that high-risk patients with diffuse histology characteristic presented the worst prognosis than other patients (Figure 9D, *p* < 0.001), as well as high-risk patients belonged to EMT subtype (Figure 9E, *p* < 0.001), therefore, the nine-consensus prognostic gene signature is a powerful addition to identify the gastric patients with poor prognosis.

## Discussion

Precision treatment for cancer patients is increasingly important, and several studies have also reported personalized therapeutic strategies in GC. The Cancer Genome Atlas Research Network reported the comprehensive molecular characterization of GC, divided into positive for Epstein–Barr virus, microsatellite unstable, genomically stable and chromosomal instability subtypes (The Cancer Genome Atlas Research Network, 2014). Zhou et al. (2020) revealed immune activation subgroups among GC patients. The immune activation subtype was associated with favorable survival and benefited more from anti-PD-1 therapy. The immunosuppressive subtype featured high immune infiltration, stromal enrichment, and transforming growth factor (TGF)-β signaling pathway activation but failed to respond to checkpoint blockade therapy, which might be suitable for the treatment of anti-PD-L1 plus anti-TGF-β together. Hu et al. (2021)identified a bipartite GC subtyping by the comprehensive analysis of multiomics data. CS1 contains the activated extracellular biological process, CS2 contains the activation of cell cycle-associated pathways, and the CS1 group has a shorter overall survival time. Several specific pathway-generated signatures also provide the prospect to predict the prognosis of GC patients, including pyroptosis (Shao et al., 2021), hypoxia (Liu et al., 2020), glycolysis (Yu et al., 2020), autophagy (Qiu et al., 2020), stemness characteristics (Mao et al., 2021), lncRNAs (Nie et al., 2020), miRNAs (Zhang et al., 2019), and m6A genes (Wu et al., 2020).

Most of the published classifiers and signatures generated based on the TCGA-STAD cohort only focus on specific pathways or gene sets. In the current study, we used GSE15459 and GSE62254 to first filter the cross-platform prognostic genes and then used LASSO Cox analysis to reduce the dimensionality of these genes, identified nine pivotal GC prognostic genes, and further generated the nine-consensus-prognostic-gene signature. We also evaluated the protein levels of the nine candidates by the Human Protein Atlas database and obtained consistent comparison results between normal *vs*. tumor and expression levels and prognosis.

The biological function of the nine genes has also been reported by several studies. Cui et al. (2020)reported that elevated NOTCH3 levels lead to poor prognosis in GC patients, and high NOTCH3 expression results in lower infiltration of activated CD8^+^ T cells and higher infiltration of Treg and M2 macrophages. Kang et al. (2021)identified that NOTCH3 can upregulate the expression of PHLDB2 and activate the AKT pathway to promote the carcinogenesis of GC.Wu et al. (2018) confirmed that the MATN3 protein levels in GC tissues were high compared to normal tissues *via* IHC staining, and the high protein level of MATN3 was associated with markedly decreased OS compared to patients with low protein levels. For MPDZ, Tetzlaff et al. (2018)reported that MPDZ can physically interact with the intracellular carboxytermini of DLL1 and DLL4, further interact with the adherent junction protein Nectin-2, and promote the activation of Notch signaling. In pancancer analyses, ANKRD6 was reported to be linked with poor prognosis and increased infiltration of M2 macrophages (Bai et al., 2021), while Yu et al. (2014)demonstrated that a significant association was observed between ANKRD6 overexpression and TNM stage, nodal metastasis and triple-negative status of breast cancer. Knockdown of ANKRD6 can decrease cell proliferation and invasion and might through the phosphorylation regulation of JNK. Few studies have focused on the biological functions of PRICKLE2, PDE12, RBP1, THSD7B, and TUBB6 in gastric cancer.

In the current study, the nine-consensus-prognostic-gene signature shows a wonderful prognostic value to identify OS results, with an AUC value as high as 0.812 in the training GSE15459 cohort and time AUC values of 0.620–0.723 in the validation and external validation cohorts, which is better than several proposed signatures (Song et al., 2017; Chen et al., 2021; Huo et al., 2021a; Huo et al., 2021b). In addition, we revealed that the nine-consensus-prognostic-gene signature is an independent prognostic factor after adjusting for clinical features and presented preferable prognostic value in different subgroups of GC patients. Moreover, the combined prognostic value of the signature and other clinical features increased to 0.860. We constructed the nomogram using only tumor stage and the nine-consensus-prognostic-gene signature, which also displayed good consistency of the nomogram-predicted events and actual events at 1, 3, and 5 years. Several nomograms have also been constructed and published for gastric cancer. Jeong et al. (2020)reported a nomogram composed of age, sex, and the expression levels of CAPZA, PPase, OCT-1, PRDX4, gamma-enolase, and c-Myc; Zhu et al. (2020)illustrated a nomogram *via* eight independent variables based on the SEER database, including race, grade, surgery, chemotherapy, and metastases of bone, brain, liver, lung; Dong et al. (2019)reported an individualized nomogram incorporating the primary tumor, peritoneum region and Lauren type recognized by computed tomography to evaluate occult peritoneal metastasis in patients with advanced gastric cancer. We hope the nomogram generated in the current study by the nine-consensus-prognostic-gene signature and tumor stage can provide novel insight for the prognostic prediction of GC patients along with the already proposed nomograms.

We observed the activation of apical junction, epithelial mesenchymal transition, WNT/beta-catenin signaling, immune associated pathways and higher immunocyte infiltration in the poor prognosis high-risk group. Epithelial-mesenchymal transition (EMT) is an important fundamental process in embryogenesis, wound healing, and fibrotic diseases (Thiery et al., 2009). Abnormal activation of EMT also plays an important role in the development, invasion and metastasis of gastric cancer (Zhao et al., 2013; Yue et al., 2019; Lin H. et al., 2021). Tian et al. reported that SERPINH1 can regulate EMT and GC progression *via* the Wnt/β-catenin pathway. Wu et al. (2021)reported that lncRNA SNHG11 can promote GC progression by activating the WNT/beta-catenin pathway. Regarding immune infiltration and GC prognosis, Jiang et al. (2021)separated GC into 3 immune cell infiltration clusters using unsupervised clustering based on the ESTIMATE and CIBERSORT algorithms, and cluster 3 with high immunocyte infiltration also presented the worst prognosis. Morihiro et al. (2019)also reported that a high protein level of PD-L1 was an independent poor prognostic factor. In the country, Kemi et al. (2020)developed the immune cell score (ICS) based on the infiltration of CD3^+^ lymphocytes and CD8^+^ lymphocytes, and the 5-years survival was 41.6% in the high ICS group, 31.7% in the intermediate ICS group and 22.2% in the low ICS group. This contradiction might be caused by the different activation statuses of the tumor microenvironment, as Zhou et al. (202) and Sato et al. (2020)reported that the immune-hot or immune-activated status inhibits tumor progression with a favorable prognosis, while the immune-cold or immunosuppressed group has a poor prognosis.

## Conclusion

Collectively, we constructed a robust nine-consensus-prognostic-gene signature for the prediction of GC prognosis. With validation in multiple cohorts, the nine-consensus-prognostic-gene signature and nomogram independently, quantitatively, and accurately predicted patient clinical outcomes. In addition, the signature also proved to be a reliable indicator of personalized treatment of GC patients.

## Data Availability

The datasets presented in this study can be found in online repositories. The names of the repository/repositories and accession number(s) can be found in the article/Supplementary Material.

## References

[B1] Bai R. Wu D. Shi Z. Hu W. Li J. Chen Y. (2021). Pan-cancer analyses demonstrate that ANKRD6 is associated with a poor prognosis and correlates with M2 macrophage infiltration in colon cancer. Chin. J. Cancer Res. 33 (1), 93–102. 10.21147/j.issn.1000-9604.2021.01.10 33707932 PMC7941690

[B2] Bang Y. J. Van Cutsem E. Feyereislova A. Chung H. C. Shen L. Sawaki A. (2010). Trastuzumab in combination with chemotherapy versus chemotherapy alone for treatment of HER2-positive advanced gastric or gastro-oesophageal junction cancer (ToGA): a phase 3, open-label, randomised controlled trial. Lancet 376 (9742), 687–697. 10.1016/S0140-6736(10)61121-X 20728210

[B3] Bang Y. J. Kim Y. W. Yang H. K. Chung H. C. Park Y. K. Lee K. H. (2012). Adjuvant capecitabine and oxaliplatin for gastric cancer after D2 gastrectomy (CLASSIC): a phase 3 open-label, randomised controlled trial. Lancet 379 (9813), 315–321. 10.1016/S0140-6736(11)61873-4 22226517

[B4] Bray F. Ferlay J. Soerjomataram I. Siegel R. L. Torre L. A. Jemal A. (2018). Global cancer statistics 2018: GLOBOCAN estimates of incidence and mortality worldwide for 36 cancers in 185 countries. CA. Cancer J. Clin. 68 (6), 394–424. 10.3322/caac.21492 30207593

[B5] Chen T. Yang C. Dou R. Xiong B. (2021). Identification of a novel 10 immune-related genes signature as a prognostic biomarker panel for gastric cancer. Cancer Med. 10 (18), 6546–6560. 10.1002/cam4.4180 34382341 PMC8446556

[B6] Cho J. Y. Lim J. Y. Cheong J. H. Park Y. Y. Yoon S. L. Kim S. M. (2011). Gene expression signature-based prognostic risk score in gastric cancer. Clin. Cancer Res. 17 (7), 1850–1857. 10.1158/1078-0432.CCR-10-2180 21447720 PMC3078023

[B7] Cristescu R. Lee J. Nebozhyn M. Kim K. M. Ting J. C. Wong S. S. (2015). Molecular analysis of gastric cancer identifies subtypes associated with distinct clinical outcomes. Nat. Med. 21 (5), 449–456. 10.1038/nm.3850 25894828

[B8] Cui Y. Li Q. Li W. Wang Y. Lv F. Shi X. (2020). NOTCH3 is a prognostic factor and is correlated with immune tolerance in gastric cancer. Front. Oncol. 10, 574937. 10.3389/fonc.2020.574937 33479597 PMC7814877

[B9] Dong D. Tang L. Li Z. Y. Fang M. J. Gao J. B. Shan X. H. (2019). Development and validation of an individualized nomogram to identify occult peritoneal metastasis in patients with advanced gastric cancer. Ann. Oncol. 30 (3), 431–438. 10.1093/annonc/mdz001 30689702 PMC6442651

[B10] Hoshida Y. Brunet J. P. Tamayo P. Golub T. R. Mesirov J. P. (2007). Subclass mapping: identifying common subtypes in independent disease data sets. PLoS One 2 (11), e1195. 10.1371/journal.pone.0001195 18030330 PMC2065909

[B11] Hu X. Wang Z. Wang Q. Chen K. Han Q. Bai S. (2021). Molecular classification reveals the diverse genetic and prognostic features of gastric cancer: a multi-omics consensus ensemble clustering. Biomed. Pharmacother. 144, 112222. 10.1016/j.biopha.2021.112222 34607103

[B12] Huo J. Wu L. Zang Y. (2021). Eight-gene prognostic signature associated with hypoxia and ferroptosis for gastric cancer with general applicability. Epigenomics 13 (11), 875–890. 10.2217/epi-2020-0411 33942671

[B13] Huo J. Wu L. Zang Y. (2021). Development and validation of a robust immune-related prognostic signature for gastric cancer. J. Immunol. Res. 2021, 5554342. 10.1155/2021/5554342 34007851 PMC8110424

[B14] Jeong S. H. Kim R. B. Park S. Y. Park J. Jung E. J. Ju Y. T. (2020). Nomogram for predicting gastric cancer recurrence using biomarker gene expression. Eur. J. Surg. Oncol. 46 (1), 195–201. 10.1016/j.ejso.2019.09.143 31564475

[B15] Jiang Q. Sun J. Chen H. Ding C. Tang Z. Ruan Y. (2021). Establishment of an immune cell infiltration score to help predict the prognosis and chemotherapy responsiveness of gastric cancer patients. Front. Oncol. 11, 650673. 10.3389/fonc.2021.650673 34307129 PMC8299334

[B16] Kang W. Zhang J. Huang T. Zhou Y. Wong C. C. Chan R. C. K. (2021). NOTCH3, a crucial target of miR-491-5p/miR-875-5p, promotes gastric carcinogenesis by upregulating PHLDB2 expression and activating Akt pathway. Oncogene 40 (9), 1578–1594. 10.1038/s41388-020-01579-3 33452458 PMC7932926

[B17] Keenan T. E. Burke K. P. Van Allen E. M. (2019). Genomic correlates of response to immune checkpoint blockade. Nat. Med. 25 (3), 389–402. 10.1038/s41591-019-0382-x 30842677 PMC6599710

[B18] Kemi N. Hiltunen N. Vayrynen J. P. Pohjanen V. M. Helminen O. Junttila A. (2020). Immune cell infiltrate and prognosis in gastric cancer. Cancers (Basel) 12 (12), E3604. 10.3390/cancers12123604 33276550 PMC7761548

[B19] Lee I. S. Lee H. Hur H. Kanda M. Yook J. H. Kim B. S. (2021). Transcriptomic profiling identifies a risk stratification signature for predicting peritoneal recurrence and micrometastasis in gastric cancer. Clin. Cancer Res. 27 (8), 2292–2300. 10.1158/1078-0432.CCR-20-3835 33558424 PMC8103893

[B20] Li Y. Liu C. Zhang X. Huang X. Liang S. Xing F. (2022). CCT5 induces epithelial-mesenchymal transition to promote gastric cancer lymph node metastasis by activating the Wnt/β-catenin signalling pathway. Br. J. Cancer 126 (12), 1684–1694. 10.1038/s41416-022-01747-0 35194191 PMC9174209

[B21] Li M. Rao X. Cui Y. Zhang L. Li X. Wang B. (2022). The keratin 17/YAP/IL6 axis contributes to E-cadherin loss and aggressiveness of diffuse gastric cancer. Oncogene 41 (6), 770–781. 10.1038/s41388-021-02119-3 34845376

[B22] Lin Y. Pan X. Zhao L. Yang C. Zhang Z. Wang B. (2021). Immune cell infiltration signatures identified molecular subtypes and underlying mechanisms in gastric cancer. NPJ Genom. Med. 6 (1), 83. 10.1038/s41525-021-00249-x 34635662 PMC8505616

[B23] Lin H. Weng J. Mei H. Zhuang M. Xiao X. Du F. (2021). 5-Lipoxygenase promotes epithelial-mesenchymal transition through the ERK signaling pathway in gastric cancer. J. Gastroenterol. Hepatol. 36 (2), 455–466. 10.1111/jgh.15184 32667711

[B24] Liu Y. Wu J. Huang W. Weng S. Wang B. Chen Y. (2020). Development and validation of a hypoxia-immune-based microenvironment gene signature for risk stratification in gastric cancer. J. Transl. Med. 18 (1), 201. 10.1186/s12967-020-02366-0 32410620 PMC7226948

[B25] Lou S. Meng F. Yin X. Zhang Y. Han B. Xue Y. (2021). Comprehensive characterization of RNA processing factors in gastric cancer identifies a prognostic signature for predicting clinical outcomes and therapeutic responses. Front. Immunol. 12, 719628. 10.3389/fimmu.2021.719628 34413861 PMC8369824

[B26] Lu X. Jiang L. Zhang L. Zhu Y. Hu W. Wang J. (2019). Immune signature-based subtypes of cervical squamous cell carcinoma tightly associated with human papillomavirus type 16 expression, molecular features, and clinical outcome. Neoplasia 21 (6), 591–601. 10.1016/j.neo.2019.04.003 31055200 PMC6658934

[B27] Lu X. Meng J. Zhou Y. Jiang L. Yan F. (2020). MOVICS: an R package for multi-omics integration and visualization in cancer subtyping. Bioinformatics 36, 5539–5541. 10.1093/bioinformatics/btaa1018 33315104

[B28] Mao D. Zhou Z. Song S. Li D. He Y. Wei Z. (2021). Identification of stemness characteristics associated with the immune microenvironment and prognosis in gastric cancer. Front. Oncol. 11, 626961. 10.3389/fonc.2021.626961 33747944 PMC7966731

[B29] McGranahan N. Furness A. J. Rosenthal R. Ramskov S. Lyngaa R. Saini S. K. (2016). Clonal neoantigens elicit T cell immunoreactivity and sensitivity to immune checkpoint blockade. Science 351 (6280), 1463–1469. 10.1126/science.aaf1490 26940869 PMC4984254

[B30] Meng J. Zhou Y. Lu X. Bian Z. Chen Y. Zhou J. (2021). Immune response drives outcomes in prostate cancer: implications for immunotherapy. Mol. Oncol. 15 (5), 1358–1375. 10.1002/1878-0261.12887 33338321 PMC8096785

[B31] Morihiro T. Kuroda S. Kanaya N. Kakiuchi Y. Kubota T. Aoyama K. (2019). PD-L1 expression combined with microsatellite instability/CD8+ tumor infiltrating lymphocytes as a useful prognostic biomarker in gastric cancer. Sci. Rep. 9 (1), 4633. 10.1038/s41598-019-41177-2 30874607 PMC6420501

[B32] Nie K. Deng Z. Zheng Z. Wen Y. Pan J. Jiang X. (2020). Identification of a 14-lncRNA signature and construction of a prognostic nomogram predicting overall survival of gastric cancer. DNA Cell Biol. 39 (9), 1532–1544. 10.1089/dna.2020.5565 32644844

[B33] Oh S. C. Sohn B. H. Cheong J. H. Kim S. B. Lee J. E. Park K. C. (2018). Clinical and genomic landscape of gastric cancer with a mesenchymal phenotype. Nat. Commun. 9 (1), 1777. 10.1038/s41467-018-04179-8 29725014 PMC5934392

[B34] Qiu J. Sun M. Wang Y. Chen B. (2020). Identification and validation of an individualized autophagy-clinical prognostic index in gastric cancer patients. Cancer Cell Int. 20, 178. 10.1186/s12935-020-01267-y 32477008 PMC7240997

[B35] Sasako M. Inoue M. Lin J. T. Khor C. Yang H. K. Ohtsu A. (2010). Gastric cancer working group report. Jpn. J. Clin. Oncol. 40 (1), i28–37. 10.1093/jjco/hyq124 20870917

[B36] Sato Y. Wada I. Odaira K. Hosoi A. Kobayashi Y. Nagaoka K. (2020). Integrative immunogenomic analysis of gastric cancer dictates novel immunological classification and the functional status of tumor-infiltrating cells. Clin. Transl. Immunol. 9 (10), e1194. 10.1002/cti2.1194 PMC7568758 33101677

[B37] Shao W. Yang Z. Fu Y. Zheng L. Liu F. Chai L. (2021). The pyroptosis-related signature predicts prognosis and indicates immune microenvironment infiltration in gastric cancer. Front. Cell Dev. Biol. 9, 676485. 10.3389/fcell.2021.676485 34179006 PMC8226259

[B38] Sohn B. H. Hwang J. E. Jang H. J. Lee H. S. Oh S. C. Shim J. J. (2017). Clinical significance of four molecular subtypes of gastric cancer identified by the cancer genome Atlas Project. Clin. Cancer Res. 23 (15), 4441–4449. 10.1158/1078-0432.CCR-16-2211 28747339 PMC5785562

[B39] Song P. Jiang B. Liu Z. Ding J. Liu S. Guan W. (2017). A three-lncRNA expression signature associated with the prognosis of gastric cancer patients. Cancer Med. 6 (6), 1154–1164. 10.1002/cam4.1047 28444881 PMC5463065

[B40] Subramanian A. Tamayo P. Mootha V. K. Mukherjee S. Ebert B. L. Gillette M. A. (2005). Gene set enrichment analysis: a knowledge-based approach for interpreting genome-wide expression profiles. Proc. Natl. Acad. Sci. U. S. A. 102 (43), 15545–15550. 10.1073/pnas.0506580102 16199517 PMC1239896

[B41] Tanaka H. Yoshii M. Imai T. Tamura T. Toyokawa T. Muguruma K. (2021). Clinical significance of coexisting histological diffuse type in stage II/III gastric cancer. Mol. Clin. Oncol. 15 (5), 234. 10.3892/mco.2021.2397 34650801 PMC8506663

[B42] Tang S. Lin L. Cheng J. Zhao J. Xuan Q. Shao J. (2020). The prognostic value of preoperative fibrinogen-to-prealbumin ratio and a novel FFC score in patients with resectable gastric cancer. BMC Cancer 20 (1), 382. 10.1186/s12885-020-06866-6 32375697 PMC7201974

[B43] Tay S. T. Leong S. H. Yu K. Aggarwal A. Tan S. Y. Lee C. H. (2003). A combined comparative genomic hybridization and expression microarray analysis of gastric cancer reveals novel molecular subtypes. Cancer Res. 63 (12), 3309–3316. 12810664

[B44] Tetzlaff F. Adam M. G. Feldner A. Moll I. Menuchin A. Rodriguez-Vita J. (2018). MPDZ promotes DLL4-induced Notch signaling during angiogenesis. Elife 7, e32860. 10.7554/eLife.32860 29620522 PMC5933922

[B45] The Cancer Genome Atlas Research Network (2014). Comprehensive molecular characterization of gastric adenocarcinoma. Nature 513 (7517), 202–209. 10.1038/nature13480 25079317 PMC4170219

[B46] Thiery J. P. Acloque H. Huang R. Y. Nieto M. A. (2009). Epithelial-mesenchymal transitions in development and disease. Cell 139 (5), 871–890. 10.1016/j.cell.2009.11.007 19945376

[B47] Thomassen I. van Gestel Y. R. van Ramshorst B. Luyer M. D. Bosscha K. Nienhuijs S. W. (2014). Peritoneal carcinomatosis of gastric origin: a population-based study on incidence, survival and risk factors. Int. J. Cancer 134 (3), 622–628. 10.1002/ijc.28373 23832847

[B48] Wu P. L. He Y. F. Yao H. H. Hu B. (2018). Martrilin-3 (MATN3) overexpression in gastric adenocarcinoma and its prognostic significance. Med. Sci. Monit. 24, 348–355. 10.12659/msm.908447 29343680 PMC5784332

[B49] Wu X. Zhang X. Tao L. Dai X. Chen P. (2020). Prognostic value of an m6A RNA methylation regulator-based signature in patients with hepatocellular carcinoma. Biomed. Res. Int. 2020, 2053902. 10.1155/2020/2053902 32733931 PMC7378627

[B50] Wu Q. Ma J. Wei J. Meng W. Wang Y. Shi M. (2021). lncRNA SNHG11 promotes gastric cancer progression by activating the wnt/β-catenin pathway and oncogenic autophagy. Mol. Ther. 29 (3), 1258–1278. 10.1016/j.ymthe.2020.10.011 33068778 PMC7934455

[B51] Yang W. Soares J. Greninger P. Edelman E. J. Lightfoot H. Forbes S. (2013). Genomics of Drug sensitivity in cancer (GDSC): a resource for therapeutic biomarker discovery in cancer cells. Nucleic Acids Res. 41, D955–D961. 10.1093/nar/gks1111 23180760 PMC3531057

[B52] Yoshihara K. Shahmoradgoli M. Martínez E. Vegesna R. Kim H. Torres-Garcia W. (2013). Inferring tumour purity and stromal and immune cell admixture from expression data. Nat. Commun. 4, 2612. 10.1038/ncomms3612 24113773 PMC3826632

[B53] Yu X. Wang M. Dong Q. Jin F. (2014). Diversin is overexpressed in breast cancer and accelerates cell proliferation and invasion. PLoS One 9 (5), e98591. 10.1371/journal.pone.0098591 24858714 PMC4032268

[B54] Yu S. Hu C. Cai L. Du X. Lin F. Yu Q. (2020). Seven-gene signature based on glycolysis is closely related to the prognosis and tumor immune infiltration of patients with gastric cancer. Front. Oncol. 10, 1778. 10.3389/fonc.2020.01778 33072557 PMC7531434

[B55] Yue B. Song C. Yang L. Cui R. Cheng X. Zhang Z. (2019). METTL3-mediated N6-methyladenosine modification is critical for epithelial-mesenchymal transition and metastasis of gastric cancer. Mol. Cancer 18 (1), 142. 10.1186/s12943-019-1065-4 31607270 PMC6790244

[B56] Zhang Z. Dong Y. Hua J. Xue H. Hu J. Jiang T. (2019). A five-miRNA signature predicts survival in gastric cancer using bioinformatics analysis. Gene 699, 125–134. 10.1016/j.gene.2019.02.058 30849543

[B57] Zhao L. Li W. Zang W. Liu Z. Xu X. Yu H. (2013). JMJD2B promotes epithelial-mesenchymal transition by cooperating with beta-catenin and enhances gastric cancer metastasis. Clin. Cancer Res. 19 (23), 6419–6429. 10.1158/1078-0432.CCR-13-0254 24077348

[B58] Zhou Y. J. Zhu G. Q. Lu X. F. Zheng K. I. Wang Q. W. Chen J. N. (2020). Identification and validation of tumour microenvironment-based immune molecular subgroups for gastric cancer: immunotherapeutic implications. Cancer Immunol. Immunother. 69 (6), 1057–1069. 10.1007/s00262-020-02525-8 32100076 PMC11027667

[B59] Zhu Y. Fang X. Wang L. Zhang T. Yu D. (2020). A predictive nomogram for early death of metastatic gastric cancer: a retrospective study in the SEER database and China. J. Cancer 11 (18), 5527–5535. 10.7150/jca.46563 32742500 PMC7391207

