# Supplementary material for: A nine–consensus–prognostic –gene–based prognostic signature, recognizing the dichotomized subgroups of gastric cancer patients with different clinical outcomes and therapeutic strategies
Source: Front Genet. 2022 Sep 26;13:909175. doi: 10.3389/fgene.2022.909175 (PMC9550166; doi:10.3389/fgene.2022.909175)
Supplement: Supplementary file 3 [file DataSheet1.PDF]

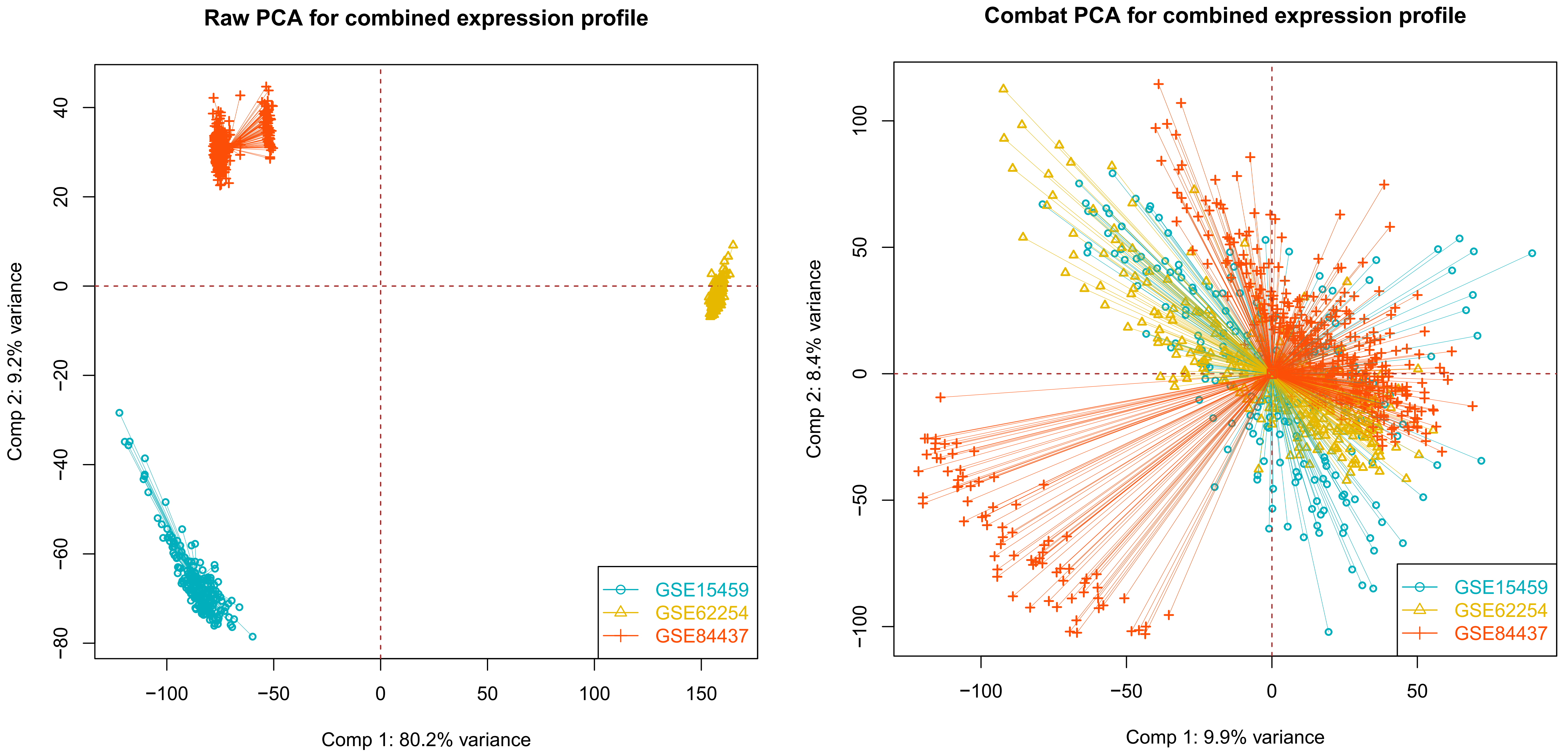

Raw PCA for combined expression profile
Comp 2: 9.2% variance
Comp 1: 80.2% variance
40
20
0
−20
−40
−60
−80
−100
−50
0
50
100
150
GSE15459
GSE62254
GSE84437
Combat PCA for combined expression profile
Comp 2: 8.4% variance
Comp 1: 9.9% variance
100
50
0
−50
−100
−100
−50
0
50
GSE15459
GSE62254
GSE84437

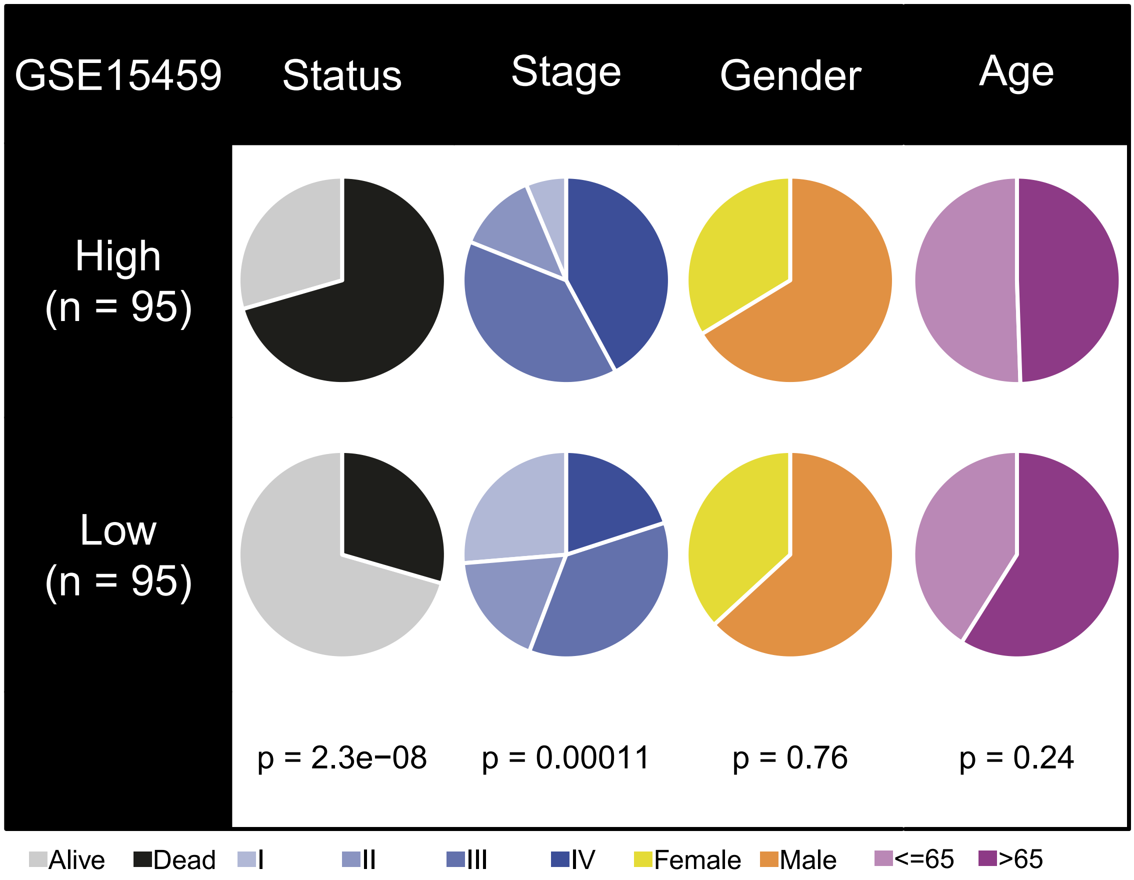
GSE15459
Status
Stage
Gender
Age
High
(n = 95)
Low
(n = 95)
p = 2.3e−08
p = 0.00011
p = 0.76
p = 0.24
Alive
Dead
I
II
III
IV
Female
Male
<=65
>65

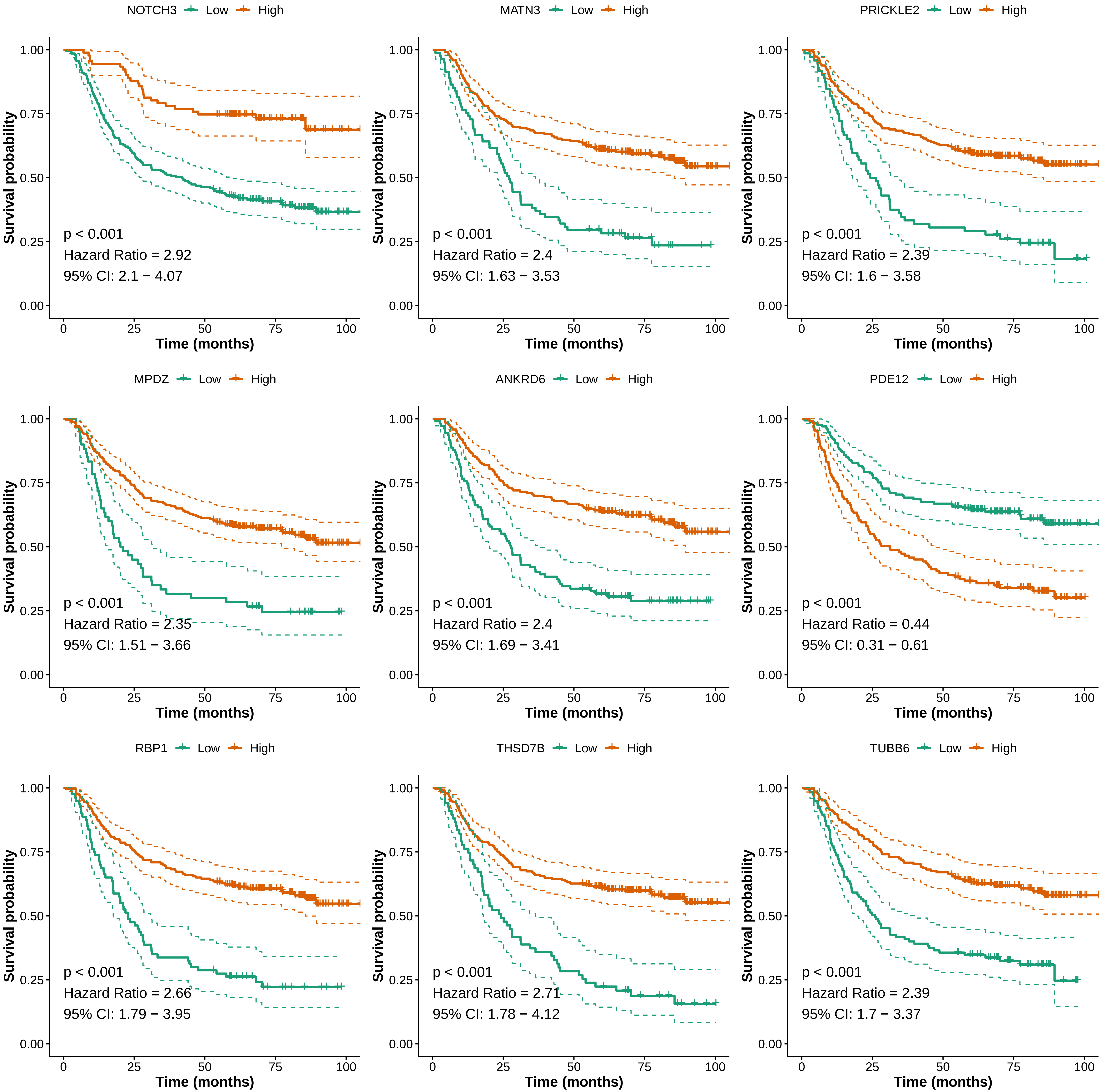
NOTCH3 Low High
Survival probability
Time (months)
p < 0.001
Hazard Ratio = 2.92
95% CI: 2.1 − 4.07
MATN3 Low High
Survival probability
Time (months)
p < 0.001
Hazard Ratio = 2.4
95% CI: 1.63 − 3.53
PRICKLE2 Low High
Survival probability
Time (months)
p < 0.001
Hazard Ratio = 2.39
95% CI: 1.6 − 3.58
MPDZ Low High
Survival probability
Time (months)
p < 0.001
Hazard Ratio = 2.35
95% CI: 1.51 − 3.66
ANKRD6 Low High
Survival probability
Time (months)
p < 0.001
Hazard Ratio = 2.4
95% CI: 1.69 − 3.41
PDE12 Low High
Survival probability
Time (months)
p < 0.001
Hazard Ratio = 0.44
95% CI: 0.31 − 0.61
RBP1 Low High
Survival probability
Time (months)
p < 0.001
Hazard Ratio = 2.66
95% CI: 1.79 − 3.95
THSD7B Low High
Survival probability
Time (months)
p < 0.001
Hazard Ratio = 2.71
95% CI: 1.78 − 4.12
TUBB6 Low High
Survival probability
Time (months)
p < 0.001
Hazard Ratio = 2.39
95% CI: 1.7 − 3.37

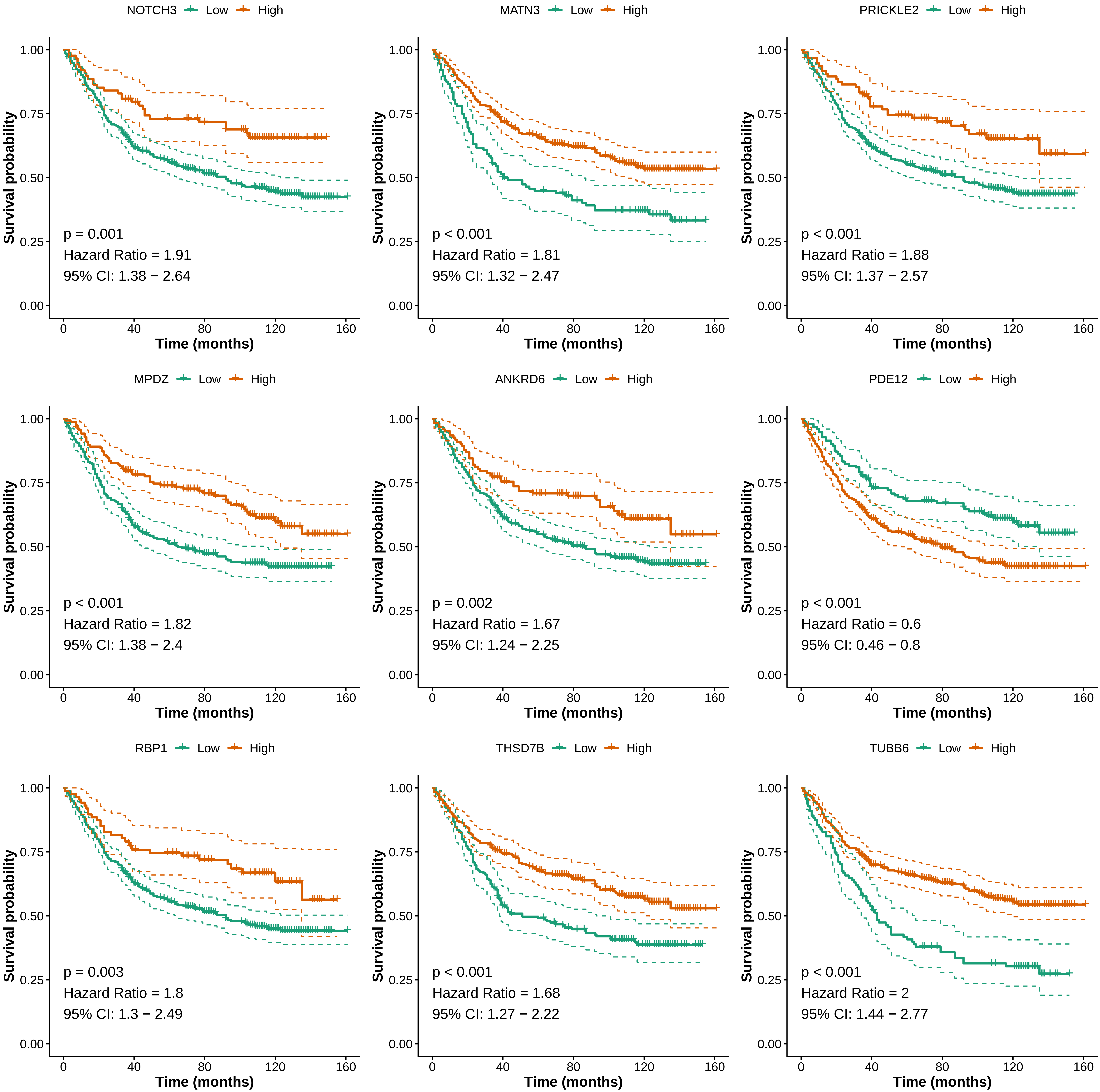

NOTCH3 Low High
p = 0.001
Hazard Ratio = 1.91
95% CI: 1.38 − 2.64
MATN3 Low High
p < 0.001
Hazard Ratio = 1.81
95% CI: 1.32 − 2.47
PRICKLE2 Low High
p < 0.001
Hazard Ratio = 1.88
95% CI: 1.37 − 2.57
MPDZ Low High
p < 0.001
Hazard Ratio = 1.82
95% CI: 1.38 − 2.4
ANKRD6 Low High
p = 0.002
Hazard Ratio = 1.67
95% CI: 1.24 − 2.25
PDE12 Low High
p < 0.001
Hazard Ratio = 0.6
95% CI: 0.46 − 0.8
RBP1 Low High
p = 0.003
Hazard Ratio = 1.8
95% CI: 1.3 − 2.49
THSD7B Low High
p < 0.001
Hazard Ratio = 1.68
95% CI: 1.27 − 2.22
TUBB6 Low High
p < 0.001
Hazard Ratio = 2
95% CI: 1.44 − 2.77
Survival probability
1.00
0.75
0.50
0.25
0.00
Time (months)
0
40
80
120
160
